# From simultaneous to leader–follower play in direct reciprocity

**DOI:** 10.1093/pnasnexus/pgag005

**Published:** 2026-01-13

**Authors:** Philip LaPorte, Lenz Pracher, Saptarshi Pal

**Affiliations:** Department of Mathematics, University of California, 970 Evans Hall, MC 3840, Berkeley, CA 94720, USA; Arnold Sommerfeld Center for Theoretical Physics, Ludwig-Maximilians-Universität München, Theresienstraße 37, München 80333, Germany; Department of Physics, Technische Universität München, James-Franck-Straße 1, Garching 85748, Germany; Department of Mathematics, Harvard University, 1 Oxford Street, Cambridge, MA 02138, USA

**Keywords:** evolutionary game theory, direct reciprocity, repeated games, Nash equilibria

## Abstract

Repeated interaction is a key mechanism driving the evolution of cooperation, generosity, extortion, and other social behaviors. These behaviors are typically studied using two-player repeated games, where the two players act simultaneously in every round. In this article, we begin with a standard two-action repeated game, but transform it into a leader–follower game. We do this by forcing one player to indicate their impending move, or to openly commit first in each round. We show that this simple transformation provides new and useful analytical insights. In particular, we use this transformation to derive expressions for the main classes of simultaneous-move Nash equilibria of memory-1, when payoffs are discounted. We show that these equilibria also remain stable under the leader–follower structure. We prove that this reassuring property does not always extend to games with more than two actions or to equilibrium strategies with longer memory. Overall, we establish a novel connection between repeated simultaneous-move games and repeated leader–follower games in evolutionary game theory. This approach offers fruitful means of investigating how prediction and anticipation play a role in stable social behavior.

Significance Statement Repeated games are important models of long-run social relationships. They involve recurring interactions between two players. Repeated games can be studied mathematically to investigate the evolution of cooperation and other social phenomena. In this article, we look at simple equilibrium outcomes of a basic repeated game. In an equilibrium, neither player has an incentive to unilaterally change behavior. We change the interaction structure so that one player is predictable, or chooses their actions each time before the other. We show that this change does not destabilize the simplest equilibria, but it can destabilize some more complicated ones. The transformation is also a useful tool for identifying equilibria in the first place.

## Introduction

Repeated games are important models of long-run relationships (1). They involve players who interact strategically in a series of rounds, receiving payoffs in each round. These models have attracted interest in biology since the early 1980s, mainly for their relevance to the evolution of cooperation. Axelrod’s seminal computer tournaments (2) first spotlighted the intriguing properties of behavioral strategies like tit-for-tat in collective settings. Early studies of tit-for-tat and other strategies showed convincingly how cooperative reciprocal behavior—direct reciprocity—might emerge under natural selection (3–6). Repeated games became established as a pillar of the evolution of cooperation literature (7).

Over the decades, the framework of repeated games has seen a number of methodological developments which have proven useful for evolutionary studies. For example, restricted strategy spaces, like the space of memory-1 strategies (5, 6, 8–11), are fruitful targets of study. These strategies decide what to do based on the previous round of interaction. They are simple enough to study computationally, but encapsulate a meaningful diversity of behavior.

Press and Dyson introduced a new perspective on memory-1 strategies by connecting payoffs with determinants (8, 10). They showed that some strategies enforce linear relationships between the long-run payoffs of players (12). A recent study found that this property also generalizes to complex settings, such as stochastic repeated games (13). Other researchers have used the Press and Dyson framework to articulate the important concepts of partner and rival strategies, and to characterize these two classes among the memory-1 strategies (14).

Another important line of development aims to study the ordering and timing of interactions in repeated games (15–21). These variables have been shown to affect the evolution of cooperation in repeated games, sometimes in important ways. To gain insight, researchers have predominantly used asynchronous repeated games (15–20). (A recent study has also examined the effect of interaction timing on the evolution of cooperation in the absence of direct reciprocity (21)).

In this article, we propose a different approach to study the effect of timing in general two-player and two-action repeated games. We begin with a conventional repeated game, in which players choose actions simultaneously in every round. We then modify it by introducing a leader–follower structure: the leader moves first in each round, and the follower moves second, with full knowledge of the leader’s choice. We develop theoretical methods that yield insights into leader–follower repeated games as well as their simultaneous-move counterparts.

In biological settings, leader–follower structures arise naturally if exogenous conditions fix the order of moves. Alternatively, the follower in a leader–follower setting may represent an individual who acquires the ability to accurately anticipate others’ (potentially randomized) moves in advance. Individuals may also develop traits, like expressive emotions (22–26) or nonverbal cues (27, 28), that force them into the role of a leader by reliably signaling their intended action. All of these mechanisms could transform a repeated simultaneous-move game into a repeated leader–follower game. We focus on this interesting transformation and what it means for equilibria.

In particular, we are motivated by the question: do Nash equilibria of repeated simultaneous games remain stable when one player assumes the position of a leader and the other assumes the position of a follower? Or, is it possible that the follower can now deviate to a more profitable strategy which takes into account an extra bit of information (the leader’s move)?

Rather than pursuing general theorems, we carry out a systematic examination of low-memory equilibria in simple two-action games. We find that, counterintuitively, the primary classes of memory-1 Nash equilibria are robust to the structural modification from simultaneous to leader–follower. However, we also show that this robustness does not hold for equilibria in even slightly more general settings. Furthermore, we discover that the transformation from the simultaneous structure to the leader–follower structure is itself a useful tool for identifying Nash equilibria of the original model, thus contributing to an existing literature within evolutionary game theory (10, 29, 30).

In the next section, we describe our model formally. Our results are organized into three parts. First, we define a notion of leader–follower stability, and we establish a useful necessary and sufficient condition for a Nash equilibrium to satisfy it. We explain how this condition can be used to search for Nash equilibria of bounded memory. Second, we show that six major classes of symmetric memory-1 Nash equilibria in two-action games are leader–follower stable. Third, we show through several examples that leader–follower stability can break down in more general settings. This includes equilibria with higher memory and memory-1 equilibria in games with more than two actions.

## Model

We consider an infinitely repeated symmetric game between two players. In each round, players simultaneously choose between two actions, labeled C and D. The outcome of each round is an element of {CC,CD,DC,DD}. Here, for example, the outcome CD means that the focal player played C and the coplayer played D. The payoff matrix


(1) $$\begin{array}{ccc} & C & D\\ C & a & b\\ D & c & d \end{array}$$

defines the payoffs for the row (focal) player at every possible outcome in a round. Following standard convention, we sometimes refer to the actions C and D as cooperate and defect. However, these labels need not reflect their usual interpretation, since the payoff parameters a,b,c,d may take arbitrary real values.

Each player follows a strategy in the repeated game. A strategy *σ* assigns a probability distribution over actions, based on the game history (see Supporting Information for formal details). We write *Σ* for the space of all possible strategies. For most of this article, we focus on a simple subset of *Σ*, the memory-1 strategies (5, 6, 8–11). A memory-1 strategy, $p=(p_{0};p_{1},p_{2},p_{3},p_{4})$, defined by five parameters, conditions its actions based only on the outcome of the previous round. Here, $p_{0}$ is the probability of playing C in the first round of the repeated game, and $p_{1},p_{2},p_{3},p_{4}$ are the probabilities of playing C if the outcome of the previous round was CC, CD, DC, or DD, respectively.

Given a strategy in *Σ* for each player, we denote by $\pi_{t}^{\left(i\right)}$ the expected payoff of player *i* in round *t*. Since strategies may involve randomization, payoffs are taken in expectation. The long-run discounted payoff of player *i* is then defined as


(2) $$(1-\delta )(\pi_{1}^{\left(i\right)}+\delta \pi_{2}^{\left(i\right)}+\delta^{2}\pi_{3}^{\left(i\right)}+\cdot \cdot \cdot ).$$

Here, δ∈(0,1) is a fixed parameter called the discount factor. It captures how players value future outcomes relative to present ones: a small value of *δ* signifies that players place much greater importance on immediate outcomes, while a value close to 1 signifies patient players who care about the average outcome in the long-run. If one player uses a strategy *σ* and the other player uses a strategy $\sigma^{\prime }$, the discounted payoff to the player who uses *σ* is written as $\pi_{\delta }(\sigma ,\sigma^{\prime })$.

A Nash equilibrium is a strategy profile $\left(\sigma ,\sigma^{\prime }\right)$ so that for all strategies $\sigma^{\prime \prime }\in \Sigma$,


(3) $$\begin{array}{c} \pi_{\delta }(\sigma ,\sigma^{\prime })\;⩾\;\pi_{\delta }(\sigma^{\prime \prime },\sigma^{\prime }),\\ \pi_{\delta }(\sigma^{\prime },\sigma )\;⩾\;\pi_{\delta }(\sigma^{\prime \prime },\sigma ). \end{array}$$

That is, neither player has an incentive to deviate to a different strategy. We are primarily interested in Nash equilibria in which players play deterministically along the equilibrium path. In other words, in the game between *σ* and $\sigma^{\prime }$, neither player ever plays C with probability other than 0 or 1. A Nash equilibrium which involves randomization between C and D at equilibrium must involve a perfect indifference between the two actions, which could be broken by a small perturbation in the parameters *a*, *b*, *c*, *d*, or *δ*. It is important, however, to note that strategies which are deterministic along the equilibrium path may behave stochastically off the path, for example, when one player deviates to an alternate strategy.

In a realistic scenario, one player may learn of the other’s imminent action in the current round before choosing their own action. This may occur if moves are no longer simultaneous but instead follow a leader–follower order, as in a Stackelberg game (31). Alternatively, this could occur if one player can reliably predict the opponent’s move from nonverbal signals such expressive emotions. In either case, the information about the coplayer’s upcoming move can be incorporated into a player’s strategy.

We wish to study this transformation from a simultaneous-move game into a leader–follower game. However, it is convenient to reflect this transformation in players’ strategies rather than explicitly in the structure of the game itself. Thus, instead of defining two separate games, a simultaneous game and a leader–follower game, we will often speak of ordinary strategies, which choose actions based on the previous rounds, and *follower-type strategies*, which additionally can observe and react to the opponent’s move in the current round, implicitly forcing the game into a leader–follower structure.

A follower-type strategy can play against an ordinary strategy. In this case, the game is implicitly a leader–follower game. The ordinary strategy is the leader in each round, choosing a move based on the previous rounds. Two ordinary strategies can also play against each other. In this case, the game is implicitly a simultaneous game. However, in general, two follower-type strategies cannot sensibly play against each other.

**Fig. 1.**
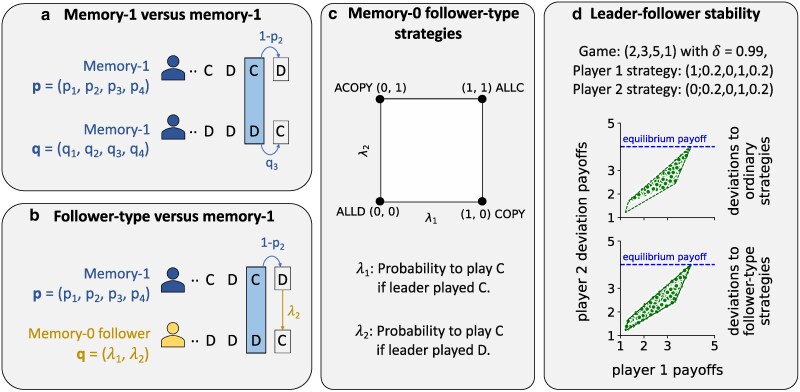
Memory-0 follower-type strategies and leader–follower stability. a) In a game between two memory-1 strategies, actions at each round are determined by the outcome of the previous round and the strategies of the two players. b) However, if a player has perfect information about their coplayer’s upcoming action (in this case, defection), they can employ a follower-type strategy that acts based on this information. The coplayer effectively becomes a leader who commits to a move first. Here, the second player adopts a memory-0 follower-type strategy that responds solely to the leader’s immediate action, ignoring all prior history. c) The space of memory-0 follower-type strategies is the unit square $[0,1{]}^{2}$, specified by two conditional probabilities: $\lambda_{1}$ and $\lambda_{2}$. These are the probabilities to cooperate if the leader’s immediate action is cooperation or defection, respectively. The deterministic strategies in this space consist of (i) the strategy that always defects, ALLD-(0,0), (ii) the strategy that always cooperates, ALLC-(1,1), (iii) the strategy that copies the leader, COPY-(1,0), and (iv) the strategy that plays the opposite of their leader’s action, ACOPY-(0,1). d) We provide an example to demonstrate a leader–follower stable Nash equilibrium. We take the repeated game G=(2,3,5,1) and consider a discount factor of δ=0.99 for the two players. The two players, labeled players 1 and 2 use the memory-1 strategies (1;0.2,0,1,0.2) and (0;0.2,0,1,0.2). In their equilibrium play, they alternate between the outcomes CD and DC. We show the payoffs that player 2 can receive if she were to make deviations towards ordinary strategies and follower-type strategies. For both cases, her payoff is always lower than, or equal to, what she attains at equilibrium. The same can be demonstrated for the first player (see Eqs. 15). Thus, this strategy profile is both a Nash equilibrium and a leader–follower stable Nash equilibrium.

For a follower-type strategy *σ*, we write σ(h,X) for the probability to play C in the current round, if the previous rounds of the game are described by the history h and the coplayer has chosen action X∈{C, D} for the current round (for a simple illustration see Fig. 1a). Ordinary strategies are a special case of follower-type strategies, where there is no dependence on X. The set of follower-type strategies is written $\Sigma_{F}$.

A *memory*-0 *follower-type strategy* is a strategy which chooses an action based only on the coplayer’s action in the current round, and not on the previous rounds. Formally, a memory-0 follower-type strategy is given by $p=(\lambda_{1},\lambda_{2})$. For an illustration, see Fig. 1b. Here, $\lambda_{1}$ is the probability to play C if the coplayer has also chosen C, and $\lambda_{2}$ is the probability to play C if the coplayer has chosen D. There are four deterministic memory-0 follower-type strategies: ALLC (1,1), which always plays C; ALLD (0,0), which always plays D; COPY (1,0), which always plays the same move as the leader; and ACOPY (0,1), which always plays the opposite move as the leader. Here, COPY and ACOPY are not to be confused with the well-known ordinary strategies tit-for-tat (TFT) and anti-tit-for-tat (ATFT), which base their next move on the opponent’s *previous* move.

One of the main questions which we address in this article is whether a Nash equilibrium in a repeated simultaneous game will survive a modification to the game structure, which allows one of the players to use a follower-type strategy. To this end, we define the notion of a leader–follower stable Nash equilibrium. We call a profile of ordinary strategies $\left(\sigma ,\sigma^{\prime }\right)$ a leader–follower-stable Nash equilibrium—or LF-stable Nash equilibrium—if, for all follower-type strategies $\sigma^{\prime \prime }\in \Sigma_{F}$, we have


(4) $$\begin{array}{c} \pi_{\delta }(\sigma ,\sigma^{\prime })\;⩾\;\pi_{\delta }(\sigma^{\prime \prime },\sigma^{\prime }),\\ \pi_{\delta }(\sigma^{\prime },\sigma )\;⩾\;\pi_{\delta }(\sigma^{\prime \prime },\sigma ). \end{array}$$

That is, neither player has an incentive to deviate to a follower-type strategy if the other player’s strategy is fixed. For an illustration, see Fig. 1c.

A trivial example of an LF-stable Nash equilibrium is a Nash equilibrium composed of deterministic strategies. A deterministic strategy never randomizes: its action is always fully determined by the previous rounds. This means that any opponent which uses a follower-type strategy, could in principle accomplish an identical result by using an ordinary strategy and looking only at the previous rounds. If neither player has a profitable deviation to an ordinary strategy, then also neither player has a profitable deviation to a follower-type strategy.

While Nash equilibria with deterministic strategies are inherently LF-stable, these equilibria are only one part of a larger picture. Many strategies important to evolutionary game theory involve stochastic components. Generous tit-for-tat (GTFT) is an example of one such strategy (5, 32–34). GTFT begins with cooperation, cooperates with probability 1 in the current round if the coplayer cooperated in the previous round, and cooperates with a nonzero probability q<1 if the coplayer defected in the previous round. When two players use GTFT, they cooperate in every round in a deterministic fashion. However, an adversarial follower-type strategy can defect against GTFT, triggering a stochastic response which can be observed before the adversary chooses its own move. By contrast, an ordinary strategy would be unable to observe the stochastic response in advance. So, it is not immediately obvious whether two strategies, like GTFT, can form an LF-stable Nash equilibrium. We will see that the general answer is nontrivial.

## Results

As we have described in Model section, allowing one player to use follower-type strategies effectively transforms a simultaneous game into a leader–follower game. Interestingly, it turns out that equilibria in the leader–follower game are easier to study, because fewer possible deviations need to be checked.

In particular, suppose $\left(\sigma ,\sigma^{\prime }\right)$ is a profile of memory-1 strategies. The property of being a Nash equilibrium means that neither player has a profitable deviation to any arbitrary strategy. However, a useful and well-known fact states that it suffices to check deviations to the 32 deterministic memory-1 strategies (35). In the special case of no discounting, or δ→1, this number can be reduced to 11 strategies, but no fewer (36).

On the other hand, the stronger property of being an LF-stable Nash equilibrium means that neither player has a profitable deviation to any follower-type strategy (which includes any ordinary strategy). Our first main result says that, surprisingly, it suffices to check just the *four* deterministic memory-0 follower-type strategies: ALLC, ALLD, COPY, and ACOPY. In other words, after checking just four conditions, we can tell whether or not a memory-1 strategy profile is an LF-stable Nash equilibrium. And if so, then it must be a Nash equilibrium as well (that is, in the simultaneous version of the game).

This result has a natural extension to memory-*n* strategy profiles as well. Briefly, a memory-*n* strategy conditions its action on the outcomes of the past *n* rounds. For a complete description of a memory-*n* strategy, one must also specify its behavior in the first *n* rounds of the game, which may depend on the ongoing game history. A memory-*n follower-type* strategy is just like a memory-*n* strategy but also conditions its behavior on the opponent’s action in the current round. We show that a strategy profile composed of two memory-*n* strategies is an LF-stable Nash equilibrium if and only if neither player has a profitable deviation to any deterministic memory-(n−1) follower-type strategy (see Proposition 2 in Methods section).

We note that there are far fewer deterministic memory-(n−1) follower-type strategies compared to deterministic memory-*n* strategies. Taking the beginning moves into account, there are $\prod_{i=0}^{n}2^{4^{i}}$ deterministic memory-*n* strategies, and only $\prod_{i=0}^{n-1}2^{2\cdot 4^{i}}$ deterministic memory-(n−1) follower-type strategies. By this measure it is $2^{(2\cdot 4^{n}+1)/3}$ times faster to check whether an arbitrary memory-*n* strategy profile is an LF-stable Nash equilibrium versus merely a Nash equilibrium. In the memory-1 case, it is 8 times faster.

In other words, while it is difficult to characterize the set of Nash equilibria of a given memory, it is comparatively much easier to characterize a useful and interesting subset: namely, those equilibria which are leader–follower stable. Accordingly, if one is checking whether a given strategy profile is a Nash equilibrium by testing all possible deviations toward deterministic strategies, it may save a considerable amount of work to first test those deterministic strategies used to verify LF-stability. If the profile is LF-stable, many unnecessary checks could be bypassed. Our result contributes to the ongoing discussion on efficient verification of Nash equilibria in direct reciprocity among bounded-memory strategies. (37, 38).

We apply these techniques to study equilibria among memory-1 strategies. In particular, we focus on memory-1 strategy profiles (p,q) which satisfy two simple conditions: (a) players have the same continuation plan, i.e. $p_{i}=q_{i}$ for i∈{1,2,3,4} and (b) players play deterministically along the equilibrium path. We adopt condition (a) because symmetric equilibria are the primary equilibria of interest for large, stable and strategically homogenous populations (9, 10, 37, 39). However, we note that our profiles need not be fully symmetric as $p_{0}$ can be different from $q_{0}$. Condition (b) excludes fragile cases where players randomize at equilibrium. Such equilibria are not robust under small changes to the game parameters.

Profiles satisfying condition (b) begin to play periodically after just a few rounds. Given condition (a), this periodic state must be either continued mutual cooperation (CC), continued mutual defection (DD), alternation between outcomes with different moves (CD, DC), alternation between outcomes with identical moves (CC, DD), or the repetition of the outcome with different moves (CD). Although there are several possibilities for the first few rounds of equilibrium play, we focus our analysis on those equilibria whose play is periodic throughout. In other words, we exclude from further analysis equilibria that achieve periodicity after a transient phase, for example, sequences which start with DD but end up in a continued CC outcome from the second round.

**Fig. 2.**
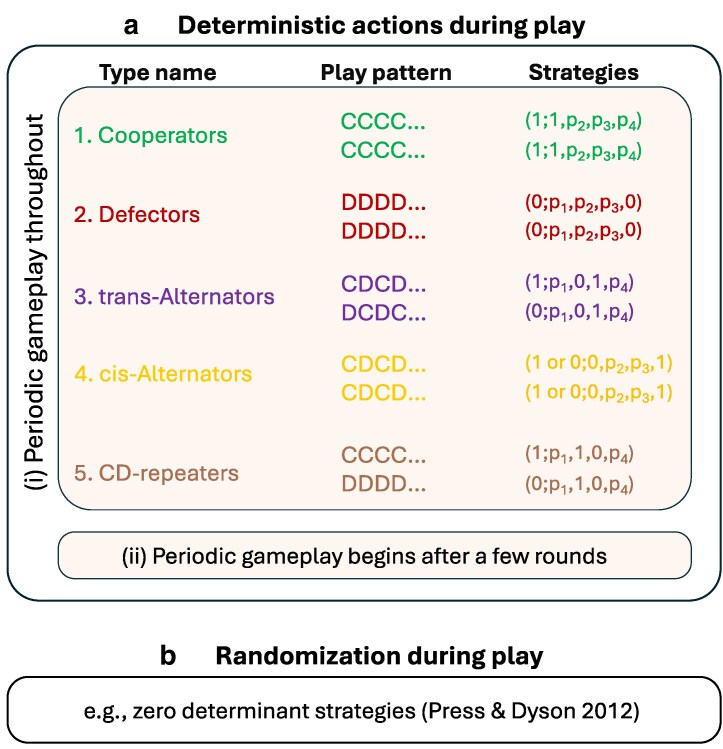
Periodic gameplay among memory-1 strategies. Consider two memory-1 strategies playing a repeated two-action game with actions C and D. We assume the two memory-1 strategies have the same continuation plan, meaning the probability with which they play C, based on the previous round, is identical. The strategies may play differently in the first round. The resulting gameplay can be broadly classified into two categories. a) In the first category, actions are taken deterministically in every round by both players. If the play is deterministic throughout, then it becomes periodic after a few moves. In the main cases we consider, the gameplay is periodic from the very first round. There are five possibilities for such gameplay: cooperators, who repeat the CC outcome; defectors, who repeat DD; trans-alternators, who alternate between CD and DC; cis-alternators, who alternate between CC and DD; and CD-repeaters, who play fixed opposite moves, repeating the outcome CD. b) In the second category, the gameplay involves randomization (for example, many zero-determinant strategies (8) are stochastic).

Thus, we study five major types of memory-1 Nash equilibria: cooperation, defection, trans-alternation, cis-alternation, and CD-repetition (see Fig. 2). In Methods section, we present the conditions on the game parameters (a,b,c,d) and the discount factor *δ* for each respective strategy profile to constitute an LF-stable Nash equilibrium. We calculate these conditions by testing deviations to the four deterministic memory-0 follower-type strategies.

Our second main result states that among these five types of memory-1 strategy profiles, there are no *additional* Nash equilibria. In other words, we prove that *all* memory-1 Nash equilibria of the five types above, are also LF-stable. Therefore, in characterizing LF-stable Nash equilibria, we have also characterized Nash equilibria in the simultaneous game. We give more detail in the next subsection.

**Fig. 3.**
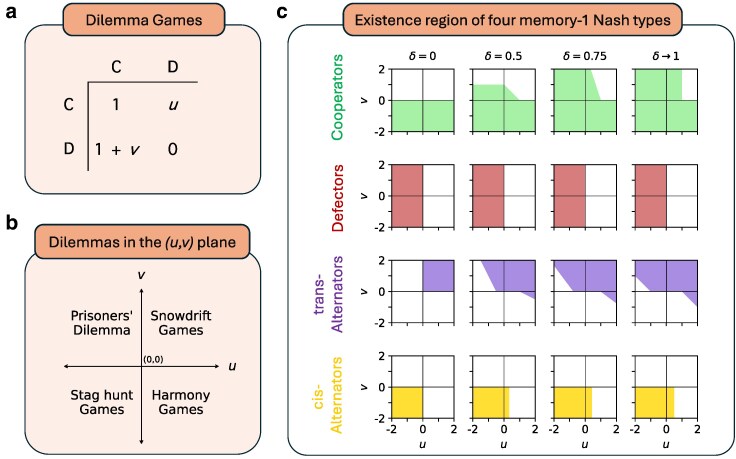
Dilemma games and the existence region for four types of memory-1 Nash equilibria. Dilemma games are those two-player, two-action, simultaneous-move games in which both players are better off at mutual cooperation than mutual defection. a) The payoffs (a,b,c,d) of a dilemma game can be expressed in terms of two parameters, *u* and *v*, as (a,b,c,d)=(1,u,1+v,0). (By normalizing with a monotonic affine-linear transformation, we can assume a=1 and d=0.) b) This defines a 2D Cartesian space of games where the four classic social dilemmas—Prisoner’s dilemma, Snowdrift, Stag hunt, and Harmony games—correspond to the four quadrants of the plane. c) We highlight regions of the (u,v)-plane where four of the five main types of memory-1 Nash equilibria—cooperators, defectors, trans-alternators, and cis-alternators—exist. These regions are plotted for four values of the discount factor: δ=0 (the one-shot game), δ=0.5, δ=0.75, and the limiting case as δ→1. For exact descriptions of the regions, see Methods section. We note that the depicted equilibria for the repeated simultaneous game are also equilibria in the associated repeated leader–follower game, with either player designated as the leader.

In Fig. 3, we depict a parameterized plane of dilemma games (games with a>d). This plane includes the Prisoner’s dilemma, Stag hunt games, Snowdrift games, and Harmony games. We show four colored regions, each of which represents the precise set of games for which a (LF-stable) Nash equilibrium type can appear. These feasibility regions change in shape as *δ* increases from 0 to 1 (see Methods section for more details).

We also examine a sixth class of equilibria often studied in evolutionary game theory: the memory-1 equalizers (8, 40). An equalizer strategy fixes any opponent’s long-run payoff at a constant value. Two equalizers form a Nash equilibrium and typically act stochastically during their equilibrium play. Expressions for equalizer strategies, known from previous studies, can be found in Methods section. We show that not only are these equilibria LF-stable, but the property of fixing the opponent’s payoff continues to work in the leader–follower setting. That is, the payoff of follower-type opponents is also fixed at the same value.

### Using follower-type strategies to study Nash equilibria

To show that all memory-1 Nash equilibria of the above five types (six including the equalizers) are in fact LF-stable, we use the elegant mathematical properties of follower-type strategies. We illustrate the technique by example.

Suppose we have a memory-1 strategy p of the form $\left(1;1;p_{2},p_{3},p_{4}\right)$ and we first wish to test whether (p,p) is an LF-stable Nash equilibrium. We must check possible deviations to four memory-0 follower-type strategies: ALLC, ALLD, COPY, and ACOPY. If either player switches to the strategy ALLC or COPY, then both players continue to cooperate. There is no change in game behavior or payoffs. So, (p,p) is an LF-stable Nash equilibrium if and only if deviations to ALLD or ACOPY are nonprofitable. That is:


(5) $$\pi_{\delta }(p,p)\;⩾\;\pi_{\delta }(\text{ALLD},p),$$

(6) $$\pi_{\delta }(p,p)\;⩾\;\pi_{\delta }(\text{ACOPY},p).$$

Simplifying these inequalities leads to a pair of analytical conditions which we report in Eq. 13 in Methods section. However, could (p,p) be a Nash equilibrium which is *not* LF-stable? Let us see why this is not in fact possible.

Suppose (p,p) meets the condition for being a Nash equilibrium. Then, it must satisfy Eq. 5 automatically: deviation to ALLD cannot be profitable for either player. This leaves Eq. 6. However, one may check that Eq. 6 is equivalent to


(7) $$\pi_{\delta }(p,p)\;⩾\;\pi_{\delta }(q,p),$$

where q is the ordinary memory-1 strategy (0;0,1,1,1). Since (p,p) is a Nash equilibrium, it meets Eq. 7 automatically, and thus Eq. 6 as well. So, (p,p) is LF-stable.

**Fig. 4.**
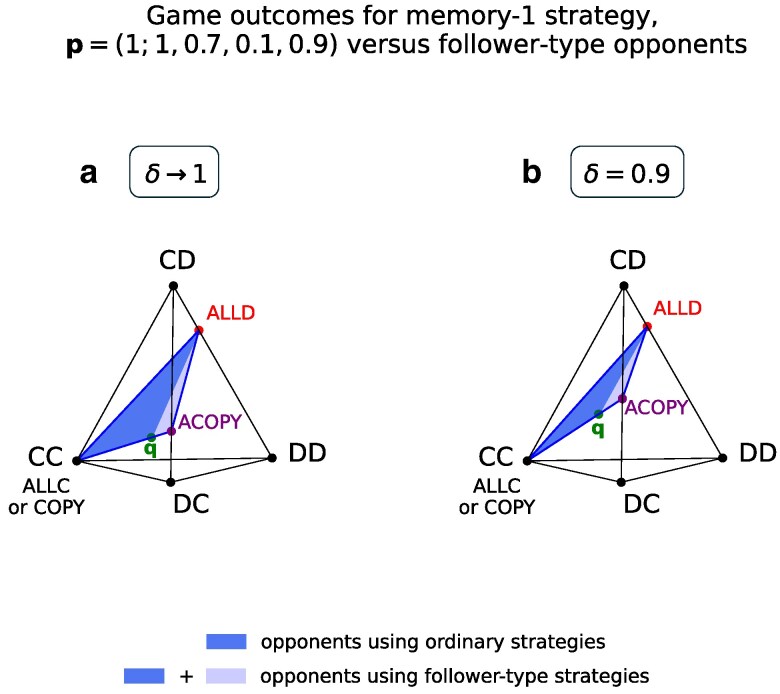
Follower-type opponents simplify the set of possible game outcomes. For this illustration, we fix an arbitrary memory-1 strategy for player 1. We have chosen p=(1;1,0.7,0.1,0.9). Next, we vary the opponent’s strategy. a) Each opponent strategy determines the long-run frequencies of the four possible outcomes CC, CD, DC, DD of each round. The frequencies determine the payoffs to both players in the limit of no discounting (δ→1). The frequencies can be plotted as a point in the standard simplex with four vertices, corresponding to the four outcomes. As the opponent varies, a subset of the simplex is traced out. Ordinary strategies, which observe only the previous rounds of the game, give rise to a 2D polygonal region, which is a triangle here with an obtuse angle labeled as q, but in general can be quite complicated. It is 2D due to a constraint known as Akin’s lemma (41), which has been also been generalized to memory-*n* strategies (37). Here, q is the memory-1 strategy (0;0,1,1,1). Follower-type strategies, which observe the opponent’s move in the current round, complete the region by extending it in all directions to the boundary of the simplex. The resulting region is simpler. Its corner points correspond to the memory-0 follower-type strategies ALLC, ALLD, COPY, and ACOPY. Notice that the point corresponding to ACOPY lies on the edge CD-DC, because ACOPY ensures that the two players make nonidentical moves in each round. The other three points also lie on their corresponding edges. b) We recreate the same plot but using the “discounted frequencies” of the four outcomes when δ=0.9 (see Supporting Information for a precise mathematical description). The discounted frequencies determine the payoff to players for the discounting case.

Of course, it was not immediately obvious that we should compare ACOPY with the memory-1 strategy q. But there is a close connection between the two, which is highlighted in Fig. 4. We depict a tetrahedron generated by the four outcomes {CC,CD,DC,DD}. A point in the tetrahedron represents a unique convex combination of these four outcomes, or, equivalently, a probability distribution over them. We use this device to plot the long-run frequencies of the four outcomes in games between p and various opponents.

Observe that the opponent q enforces that the outcome of each round belongs to the subset {CC,CD,DC} (a triangular face of the tetrahedron). On the other hand, ACOPY enforces that the outcome of each round belongs to the smaller subset {CD,DC} (the vertical edge). In effect, q can be interpreted as a mixture between p and ACOPY (see Fig. 4).

Overall, the idea is that the opponents ALLD and q give rise to necessary conditions (5) and (7) for Nash equilibrium; and the opponents ALLD and ACOPY give rise to sufficient conditions (5), (6) for LF-stable Nash equilibrium. Now, q was chosen in such a way that the two sets of conditions coincide. Therefore we know that the strategy profile is an LF-stable Nash equilibrium if and only if it is a Nash equilibrium. We also get expressions for exactly when this occurs.

A similar technique can be used for all the Nash equilibrium types which we study in this article. See Supporting Information for more details.

### Failure of leader–follower stability

The memory-1 results deceptively suggest that LF-stability is a trivial condition; perhaps that any Nash equilibrium which involves deterministic play along the equilibrium path, is also leader–follower-stable. However, we will demonstrate that this is not the case, even in slightly more general settings. We provide two examples; one involving equilibrium strategies with longer memory and one involving memory-1 strategies in a game with three actions.

The main insight is that when a Nash equilibrium involves deterministic play along the equilibrium path, a follower-type strategy may deliberately upset the equilibrium in order to provoke a stochastic response. If the response can be read, the opponent may be able to take advantage by coordinating with it in each round, in a way that is impossible with simultaneous actions. For each example below, see Supporting Information for more details and proofs.

Example 1 **(Memory-2 equilibrium in two-action game)**

Consider a repeated game in which the stage game is a Snowdrift game with the payoff matrix

(8) $$\begin{array}{ccc} & C & D\\ C & 3 & \frac{5}{2}\\ D & 5 & 1 \end{array}$$

Consider a memory-2 strategy *σ*, which plays C if neither player has played D in the previous two rounds, and otherwise plays C with probability *q* that is close to 3/7—a value which happens to make the coplayer indifferent between taking action C or action D. In the first two rounds, *σ* plays C. The strategy profile (σ,σ) is a Nash equilibrium when *δ* is large. When both players use *σ*, they always play C and each gets a total discounted payoff 3. The equilibrium is deterministic along the equilibrium path. If one player deviates and plays D, then it takes several rounds to return to a state in which neither player has played D in the previous two rounds. In the meantime, the deviating player earns less than 3 per round on average, regardless of their chosen action. When rigorous calculation is made, one sees that the deviation will not be profitable for *δ* close to 1.

On the other hand, this Nash equilibrium is not LF-stable. There is a follower-type strategy which, after two consecutive rounds of CC, plays D; and subsequently, simply plays the opposite of the opponent’s move. This strategy earns more than 3 per round in the long run.

Example 2 **(Memory-1 equilibrium in a three action game)**

We present an example which demonstrates that even memory-1 equilibria can be undermined by follower-type opponents if the game involves more than two actions. Consider a three-action game with payoff matrix

(9) $$\begin{array}{cccc} & C & D & E\\ C & 10 & -20 & -1\\ D & -20 & 10 & -1\\ E & -1 & -1 & 0 \end{array}$$

Consider the memory-1 strategy *σ* which plays E in the first round and after the outcome EE. Otherwise, *σ* plays C or D, with approximately equal probability. The strategy profile (σ,σ) is a Nash equilibrium in which both players always play E. If one player deviates to C or D, then that player earns an expected payoff that is less than or equal to −1 in every subsequent round.

On the other hand, consider the follower-type strategy which plays D in the first round and subsequently matches the opponent’s action. If one of the two players in the equilibrium (σ,σ) deviates to this strategy, they will earn 10 in every round after the first, so the deviation is profitable. The Nash equilibrium fails to be LF-stable.

Remark We emphasize that these examples are not precisely calibrated or knife-edge cases. It is straightforward to construct similar examples, with different parameters, that exhibit the same property. We give an additional example in the Supporting Information involving a cooperative memory-3 equilibrium for a donation game.

## Discussion

In this article, we study a relationship between repeated simultaneous games and repeated leader–follower games of direct reciprocity. We find that this relationship allows us to extend the boundaries of what is known in both models.

In the case of simultaneous games, we recover and augment previous work by characterizing the main classes of memory-1 equilibrium strategies in games with discounting. Our method of looking at an associated leader–follower game is demonstrated in Results section, together with the illustration in Fig. 4. We also present an extension of this method to equilibria with longer memory (see Proposition 2). The result is a computationally efficient way to confirm that certain strategy profiles are Nash equilibria. Our article complements the results of a previous study on symmetric two-player, two-action repeated games (9). In that paper, the authors report that memory-1 Nash equilibrium strategies fall into four categories. In our terminology, these are the cooperators, the defectors, the trans-alternators, and the equalizers. Our findings suggest that there is an additional category which belongs in this list: the cis-alternators.

On the other hand, our methods also show that these equilibria remain stable when simultaneity breaks, and one player is forced to be the leader in every round. The case of memory-1 strategies in two-action games is special in this regard. With higher memory or a larger set of available actions, we show that equilibria need not remain stable when players become aware of their coplayers’ upcoming move.

In a way our result lends support to the standard two-action repeated game model. This model has long been the prototype for understanding direct reciprocity (8, 10, 14, 40, 42). Notably, several key insights into direct reciprocity arise from analyzing just the stable strategies among memory-1 strategies, the simplest class of conditional strategy (5, 6, 11, 43). Our result suggests that these insights, which come from the analysis of memory-1 strategies, are robust to some degree under realistic modifications to the simultaneous game structure. Nevertheless, stable longer memory strategies should be interpreted cautiously, as their stability may hold only in the simultaneous-move model. Future research should thus investigate the defining properties of leader–follower-stable memory-*n* strategies (where n>1).

The notion of leader–follower-stability may be understood as the stability of Nash equilibria against intelligent agents capable of predicting the equilibrium strategy’s next move with high accuracy across a wide range of situations, such as deep learning systems (44) or large language models (45). It likewise applies to cases where individuals develop traits such as expressive emotions (22–26) or nonverbal cues (27, 28, 46, 47) that unintentionally reveal information about their intended move. To gain a more comprehensive understanding of leader–follower stability in complex environments, future research should seek to extend this concept to broader contexts, such as stochastic repeated games (13, 20).

Finally, our work provides a framework to systematically study the evolution of strategies in leader–follower direct reciprocity. For example, in Methods section, we present a more general model in which neither player is the permanent leader. In each round, the game is played simultaneously with probability $\rho_{0}$ or as a leader–follower game with probability $1-\rho_{0}$. When simultaneity is broken, either player 1 becomes the follower (with probability $\rho_{1}$) or player 2 becomes the follower (with probability $\rho_{2}$). This leads to $\rho_{0}+\rho_{1}+\rho_{2}=1$. We derive expected payoff expressions for both players, assuming they use memory-1 strategies when interacting simultaneously or as leaders, and memory-0 follower-type strategies when acting as followers.

**Fig. 5.**
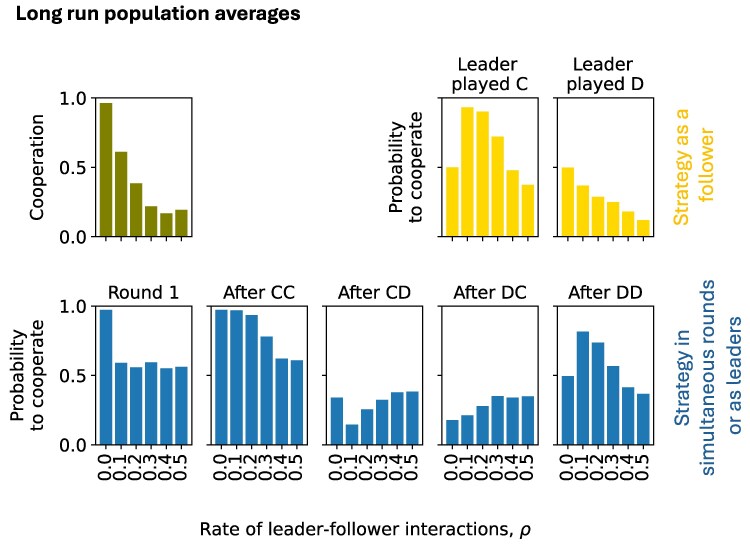
The effect of leader–follower stage games on the evolution of cooperation in a learning population: a representative example. We use simulations to examine how individual adaptation in repeated donation game strategies shapes long-run cooperation in a population. In our simulations, agents update their strategies through an imitation–exploration dynamic (see Methods section for details). The stage game is either simultaneous with probability 1−2ρ, or sequential with probability 2ρ. When sequential, both players are equally likely to lead and follow. Players use a memory-1 strategy when they play simultaneously or while leading. When they follow, they use a memory-0 follower-type strategy. We perform simulations on a population of size N=50, running the evolutionary process until a total of $T_{\mathrm{run}}=10^{8}$ strategy update events have occurred. We plot the long-run frequency of cooperation in the population (top left) and the average strategic composition of the population (top right and bottom), as we vary the parameter *ρ* from 0 (fully simultaneous) to 0.5 (fully leader–follower). Other parameters in this simulation: benefit of cooperation B=5, cost of cooperation C=1, game discount factor δ=0.99, intensity of selection during imitation β=10, rate of strategic exploration m=0.1.

A typical simulation illustrates how such interaction structures can influence behavior in evolutionary settings. We simulate a finite population of players for the repeated donation game. The players are all equivalent. In particular, $\rho_{1}=\rho_{2}=\rho$. This means the stage game in each round is simultaneous with probability 1−2ρ and sequential with the complementary probability, 2ρ. When sequential, both players are equally likely to lead. Individuals update strategies over time through imitation and exploration (please see Methods section for a complete description of the evolutionary process). In Fig. 5, we show the long-run outcomes of the learning process as *ρ* varies. Other key parameters are fixed as indicated in the caption. We find that cooperation in the donation game is highest when play is fully simultaneous (ρ=0) and declines as *ρ* increases. When play is mostly simultaneous, followers typically learn to copy the leader’s action; however, at high *ρ* values, they tend to defect regardless of the leader’s action. This leads to low levels of cooperation.

These simulations show that although cooperative equilibria exist in leader–follower settings, they may fail to emerge when players use imitation and exploration to adapt. Future work should examine these evolutionary simulations across a range of parameters for a full picture. Future studies could further investigate how longer-memory strategies for leaders and followers, leadership asymmetries, and asynchronous or individually timed updates in structured populations shape the evolution of cooperation (48–50).

## Methods

### Leader–follower-stability of memory-*n* nash equilibria

A memory-*n* strategy conditions its move in the present round based on the outcome of the previous *n* rounds. During the first *n* rounds, the moves can be chosen arbitrarily based on the previous rounds. The space of memory-*n* strategies will be denoted ${Mem}^{n}$. A memory-*n* follower-type strategy chooses a move based on the outcome of the previous rounds, together with the opponent’s choice of move in the current round. During the first *n* rounds, the moves can be chosen arbitrarily based on the previous rounds, together with the opponent’s choice of move. The space of memory-*n* follower-type strategies will be denoted ${FMem}^{n}$.

We make use of the following two propositions. The first can be found in Levínský et al. (35). The second is novel. See the Supporting Information for the proof of Proposition 2 and for more details on the methods described here.

Proposition 1 A profile of memory-*n* strategies (p,q) constitutes a Nash equilibrium if and only if the following inequalities hold for all deterministic strategies $p^{\prime },q^{\prime }\in {Mem}^{n}$:

(10) $$\begin{array}{c} \pi_{\delta }(p,q)\;⩾\;\pi_{\delta }(p^{\prime },q),\\ \pi_{\delta }(q,p)\;⩾\;\pi_{\delta }(q^{\prime },p) \end{array}$$

Proposition 2 A profile of memory-*n* strategies (p,q) constitutes an LF-stable (that is, leader–follower-stable) Nash equilibrium if and only if the following inequalities hold for all deterministic strategies $p^{\prime },q^{\prime }\in {FMem}^{n-1}$:

(11) $$\begin{array}{c} \pi_{\delta }(p,q)\;⩾\;\pi_{\delta }(p^{\prime },q),\\ \pi_{\delta }(q,p)\;⩾\;\pi_{\delta }(q^{\prime },p) \end{array}$$

Proposition 2 implies that to check memory-1 strategy profiles for LF-stability, it suffices to check that neither player has a profitable deviation to the four deterministic memory-0 follower-type strategies: ALLC, ALLD, COPY, and ACOPY.

### Formula for long-run discounted payoff

Here, we provide a simple formula to compute the long-run discounted payoffs of players when one uses the memory-0 follower-type strategy, $p=(\lambda_{1},\lambda_{2})$ and the other uses the memory-1 strategy, $q=(q_{0};q_{1},q_{2},q_{3},q_{4})$. The payoff for the p player, calculated with discount factor *δ*, is given by


(12) $$\begin{array}{c} \pi_{\delta }(p,q)=w\cdot (a,b,c,d)\\ w\;:\;=(1-\delta )v(I-\delta M{)}^{-1}\\ v\;:\;=(\lambda_{1}q_{0},\lambda_{2}(1-q_{0}),(1-\lambda_{1})q_{0},(1-\lambda_{2})(1-q_{0}))\\ M\;:=\left(\begin{array}{cccc} \lambda_{1}q_{1} & \lambda_{2}(1-q_{1}) & (1-\lambda_{1})q_{1} & \left(1-\lambda_{2})(1-q_{1}\right)\\ \lambda_{1}q_{3} & \lambda_{2}(1-q_{3}) & (1-\lambda_{1})q_{3} & \left(1-\lambda_{2})(1-q_{3}\right)\\ \lambda_{1}q_{2} & \lambda_{2}(1-q_{2}) & (1-\lambda_{1})q_{2} & \left(1-\lambda_{2})(1-q_{2}\right)\\ \lambda_{1}q_{4} & \lambda_{2}(1-q_{4}) & (1-\lambda_{1})q_{4} & \left(1-\lambda_{2})(1-q_{4}\right) \end{array}\right) \end{array}$$

Here, I is the identity matrix of size four. The payoff to q can be calculated by the same formula but with *b* and *c* switched. See Supporting Information for more details and a later section for a more general payoff formula.

### Characterization of the five classes of memory-1 nash equilibria

Here, we present the explicit existence conditions for Nash equilibria belonging to each of the five types mentioned in the main text. These are equilibria for the repeated simultaneous game with stage game payoff matrix (a,b,c,d) and discount factor *δ* for players. However, these are also LF-stable equilibria. In other words, they remain equilibria in the associated leader–follower game, with either player designated as the follower. Our results show that the two concepts coincide, for these five types. (See Supporting information for proofs.)

1. **Cooperation**: In this equilibrium, the outcome of every round is CC. This is achieved by a memory-1 strategy profile of the form $\left((1;1,p_{2},p_{3},p_{4}),(1;1,p_{2},p_{3},p_{4})\right)$. It constitutes a Nash equilibrium if and only if:
  (13) $$\begin{array}{c} (1-p_{2})(a-d)+p_{4}(a-c)\;⩾\;\left(\frac{1-\delta }{\delta }\right)(c-a),\\ (1-p_{2})(a-b)+p_{3}(a-c)\;⩾\;\left(\frac{1-\delta }{\delta }\right)(c-a). \end{array}$$
2. **Defection**: Here, the outcome of every round is DD. This is achieved by a memory-1 strategy profile of the form $\left((0;p_{1},p_{2},p_{3},0),(0;p_{1},p_{2},p_{3},0)\right)$. It constitutes a Nash equilibrium if and only if:
  (14) $$\begin{array}{c} \;p_{3}(a-d)+p_{1}(d-b)⩽\left(\frac{1}{\delta }\right)(d-b),\\ p_{3}(c-d)+p_{2}(d-b)⩽\left(\frac{1}{\delta }\right)(d-b). \end{array}$$
3. **trans-Alternation**: Here, the play begins with either CD or DC and alternates between those two outcomes from the very first round. This is achieved by a memory-1 strategy profile of the form $\left((1;p_{1},0,1,p_{4}),(0;p_{1},0,1,p_{4})\right)$. It constitutes a Nash equilibrium if and only if:
  (15) $$\begin{array}{c} \;p_{1}(c-b)⩽\left(\frac{1+\delta }{\delta }\right)(c-a),\\ (1-p_{4})(c-b)\;⩾\;\left(\frac{1+\delta }{\delta }\right)(d-b). \end{array}$$
4. **cis-Alternation**: Here, the play begins with either CC or DD and alternates between the two outcomes from the very first round. This play is achieved by memory-1 strategy profiles of the form $\left((1;0,p_{2},p_{3},1),(1;0,p_{2},p_{3},1)\right)$ or $\left((0;0,p_{2},p_{3},1),(0;0,p_{2},p_{3},1)\right)$. The conditions for these profiles to constitute a Nash equilibrium are identical. They do so if and only if:
  (16) $$\begin{array}{c} \;p_{2}(a-d)⩽\left(\frac{1+\delta }{\delta }\right)(a-c),\\ (1-p_{3})(a-d)\;⩾\;\left(\frac{1+\delta }{\delta }\right)(b-d). \end{array}$$
5. **CD-Repetition**: Here, the outcome of every round is CD. This is achieved by a memory-1 strategy profile of the form $\left((1;p_{1},1,0,p_{4}),(0;p_{1},1,0,p_{4})\right)$. It constitutes a Nash equilibrium if and only if:
  (17) $$\begin{array}{c} (1-p_{1})(b-c)⩽\left(\frac{1-\delta }{\delta }\right)(c-a),\\ p_{4}(c-b)⩽\left(\frac{1-\delta }{\delta }\right)(b-d),\\ (1-p_{1})(c-d)+p_{4}(c-a)\;⩾\;\left(\frac{1-\delta }{\delta }\right)(a-c),\\ p_{1}(d-b)+p_{4}(b-a)\;⩾\;\left(\frac{1}{\delta }\right)(d-b). \end{array}$$

Some earlier papers have studied memory-1 Nash equilibria for symmetric 2×2 games and therefore some of these expressions are not new. Expressions for cooperators and defectors have already been derived by other methods in the limiting case δ→1 (see Ref. (41)), and also for arbitrary *δ* (see Ref. (30)). Another work has also divulged expressions for the cooperators, defectors, trans-alternators and CD-repeaters in the limiting case δ→1 (see Ref. (10)). In contrast to these papers, our method yields a more comprehensive result on memory-1 Nash equilibria. The list above allows us to study the existence of these memory-1 equilibrium types for any game (a,b,c,d) and any *δ*. In Fig. 3, we have provided a useful illustration of these conditions for an important subset of 2×2 games, the dilemma games. In a dilemma game, mutual cooperation is better for both player than mutual defection: a>d. Some notable points from our illustration are as follows.

A cooperative equilibrium cannot exist if (c−a)>(δ/(1−δ))(a−d) (see also Hilbe et al. (30)). The existence of an equilibrium of defection depends on *a*, *b*, *c*, *d* but not on *δ*. If *δ* is sufficiently large, a trans-alternating equilibrium can exist for any given Prisoner’s dilemma game with b+c>2d (for example, in a donation game) and for any given game with b+d>2a. An equilibrium of cis-alternation can exist for any stag hunt game, and cannot exist if a+d<2b or if c>d.

### Characterizing memory-1 equilibria with equalizer strategies

In addition to the five major classes of memory-1 equilibria, we also study a sixth class: the equalizers. A memory-1 strategy $p=(-;p_{1},p_{2},p_{3},p_{4})$ is called an *equalizer* (51, 52) if, for any given $p_{0}$, the long-run discounted payoff to the opponent of p is independent of the opponent’s strategy. The equalizer strategies are the solutions of the system


(18) $$\frac{c-d}{1-\delta (p_{2}-p_{4})}=\frac{a-b}{1-\delta (p_{1}-p_{3})}=\frac{a-d}{1-\delta (p_{1}-p_{4})}=\frac{c-b}{1-\delta (p_{2}-p_{3})}.$$

The very last equality is redundant. Derivation of these equations, or equivalent ones, can be found in Ichinose and Masuda (52). Two equalizer strategies form a Nash equilibrium because unilateral deviations yield the same payoff as the equilibrium payoff.

In the Supporting Information, we derive these as the defining equations for *leader–follower equalizers*: the payoff to the opponent is the same even if the opponent is allowed to use follower-type strategies. That is: we show that every memory-1 equalizer strategy is a leader–follower equalizer. This is a noteworthy fact, as equalizer strategies form Nash equilibria which in general do not play deterministically along the equilibrium path.

This is an additional example of the insight provided by our methods. The conditions for ALLC, ALLD, COPY, and ACOPY to receive the same payoff are easily derived to be Eq. 18 (see Supporting Information). These elegant expressions are equivalent to the expressions for an ordinary equalizer strategy, which were previously derived in a different form and by other methods (52).

### An extended model

In the leader–follower model which we have analyzed, we assume that one player, say player 1, is always the follower. Note that player 1 is not required to always use the information of the leader’s move from the same round. Therefore, our model can be used for cases where player 1 knows the leader’s move with some probability ρ<1.

However, the model does not capture more symmetric situations in which the follower role may fall to either player with some probability in each round. In simulations, it may be useful to study leader–follower interactions in a setting where all players can access a single strategy space. To this end, we consider the following extended model:

1. Each player’s strategy has two components: the first component is an ordinary strategy (i.e. one which looks at the previous rounds only) and the second component is a follower-type strategy. That is, player 1 has $(\sigma_{1},\sigma_{1}^{\prime })\in \Sigma \times \Sigma_{F}$ and player 2 has $(\sigma_{2},\sigma_{2}^{\prime })\in \Sigma \times \Sigma_{F}$.
2. In each round, there are three mutually exclusive possibilities. With probability $\rho_{1}$, player 1 is the follower and player 2 is the leader. In this case, player 1 uses $\sigma_{1}^{\prime }$ and player 2 uses $\sigma_{2}$. In the second case, with probability $\rho_{2}⩽1-\rho_{1}$, player 2 is the follower and player 1 is the leader. In this case, player 1 uses $\sigma_{1}$ and player 2 uses $\sigma_{2}^{\prime }$. Finally, with probability $\rho_{0}=1-\rho_{1}-\rho_{2}$, neither player is the follower. In other words, simultaneity is maintained. In this case, player 1 uses $\sigma_{1}$ and player 2 uses $\sigma_{2}$.

Here, $\rho_{i}$ may be interpreted as the probability that player *i* observes the opponent’s move before choosing a move themselves. The simplest case is when both players use a pair consisting of a memory-1 strategy and a memory-0 follower-type strategy. The strategy space is then ${Mem}^{1}\times {FMem}^{0}$. Suppose player 1 and player 2 use, respectively,


(19) $$\begin{array}{c} p=(p_{0};p_{1},p_{2},p_{3},p_{4},\lambda_{1},\lambda_{2})\in {Mem}^{1}\times {FMem}^{0}\\ q=(q_{0};q_{1},q_{2},q_{3},q_{4},\mu_{1},\mu_{2})\in {Mem}^{1}\times {FMem}^{0}. \end{array}$$

Then, the Markov chain has the transition matrix


(20) $$\begin{array}{c} M=(1-\rho_{1}-\rho_{2})M_{0}+\rho_{1}M_{1}+\rho_{2}M_{2}\\ M_{0}\;:\;=\left(\begin{array}{cccc} \;p_{1}q_{1} & p_{1}(1-q_{1}) & (1-p_{1})q_{1} & \left(1-p_{1})(1-q_{1}\right)\\ p_{2}q_{3} & p_{2}(1-q_{3}) & (1-p_{2})q_{3} & \left(1-p_{2})(1-q_{3}\right)\\ p_{3}q_{2} & p_{3}(1-q_{2}) & (1-p_{3})q_{2} & \left(1-p_{3})(1-q_{2}\right)\\ p_{4}q_{4} & p_{4}(1-q_{4}) & (1-p_{4})q_{4} & \left(1-p_{4})(1-q_{4}\right) \end{array}\right)\\ M_{1}\;:\;=\left(\begin{array}{cccc} \lambda_{1}q_{1} & \lambda_{2}(1-q_{1}) & (1-\lambda_{1})q_{1} & \left(1-\lambda_{2})(1-q_{1}\right)\\ \lambda_{1}q_{3} & \lambda_{2}(1-q_{3}) & (1-\lambda_{1})q_{3} & \left(1-\lambda_{2})(1-q_{3}\right)\\ \lambda_{1}q_{2} & \lambda_{2}(1-q_{2}) & (1-\lambda_{1})q_{2} & \left(1-\lambda_{2})(1-q_{2}\right)\\ \lambda_{1}q_{4} & \lambda_{2}(1-q_{4}) & (1-\lambda_{1})q_{4} & \left(1-\lambda_{2})(1-q_{4}\right) \end{array}\right)\\ M_{2}\;:\;=\left(\begin{array}{cccc} \;p_{1}\mu_{1} & p_{1}(1-\mu_{1}) & (1-p_{1})\mu_{2} & \left(1-p_{1})(1-\mu_{2}\right)\\ p_{2}\mu_{1} & p_{2}(1-\mu_{1}) & (1-p_{2})\mu_{2} & \left(1-p_{2})(1-\mu_{2}\right)\\ p_{3}\mu_{1} & p_{3}(1-\mu_{1}) & (1-p_{3})\mu_{2} & \left(1-p_{3})(1-\mu_{2}\right)\\ p_{4}\mu_{1} & p_{4}(1-\mu_{1}) & (1-p_{4})\mu_{2} & (1-p_{4})(1-\mu_{2}). \end{array}\right) \end{array}$$

The probability distribution describing the initial moves is


(21) $$\begin{array}{c} v=(1-\rho_{1}-\rho_{2})v_{0}+\rho_{1}v_{1}+\rho_{2}v_{2}\\ v_{0}\;:\;=(p_{0}q_{0},p_{0}(1-q_{0}),(1-p_{0})q_{0},(1-p_{0})(1-q_{0}))\\ v_{1}\;:\;=(\lambda_{1}q_{0},\lambda_{2}(1-q_{0}),(1-\lambda_{1})q_{0},(1-\lambda_{2})(1-q_{0}))\\ v_{2}\;:\;=(p_{0}\mu_{1},p_{0}(1-\mu_{1}),(1-p_{0})\mu_{2},(1-p_{0})(1-\mu_{2})). \end{array}$$

The payoff to player 1 can be calculated as usual,


(22) $$\pi_{\delta }(p,q)=(1-\delta )v(I-\delta M{)}^{-1}\cdot (a,b,c,d).$$

The same approach works for games with more than two actions, or for strategies with higher memory.

### Description of the evolutionary process

We consider a population of *N* agents. The strategy of individual *i*, $\sigma_{i}\in {Mem}^{1}\times {FMem}^{0}$, is denoted by $\left(p_{0}^{i};p_{1}^{i},p_{1}^{i},p_{2}^{i},p_{3}^{i},p_{4}^{i},\lambda_{1}^{i},\lambda_{2}^{i}\right)$. For the simulation, we consider $p_{0}^{i}\in \{0,1\}$. Individuals are randomly matched in pairs to play the repeated donation game with parameters *B* and *C*. The parameter *B* denotes the benefit of cooperation and the parameter *C*, the cost of cooperation. The resulting payoff matrix is (a,b,c,d)=(B−C,−C,B,0). Players have a discount factor *δ* in the repeated game. With probability 2ρ, a given round in the repeated game proceeds sequentially, with both players equally likely to act as the follower ($\rho_{1}=\rho_{2}=\rho$). The round is simultaneous with probability 1−2ρ. The population average payoff of player *i* is denoted by $\pi^{i}$, computed assuming an equal chance of being matched with any other population member. At each strategy update step, an individual *l* is chosen at random. With probability *m*, the selected individual explores a new strategy. During exploration, individuals are equally likely to sample a new memory-1 strategy $\left(p_{0},p_{1},p_{2},p_{3},p_{4}\right)$, used in simultaneous and leader rounds, or a new memory-0 follower-type strategy $\left(\lambda_{1},\lambda_{2}\right)$, used in follower rounds. Thus, after exploration, one part of the updating individual’s strategy remains unchanged. With probability 1−m, the individual *l* instead imitates a randomly selected role model k≠l, adopting their strategy with probability $(1+\exp\;(\beta (\pi^{l}-\pi^{k}))){)}^{-1}$. Here, *β* is the intensity of imitation based on payoff differences. The evolutionary process goes on for $T_{\mathrm{run}}$ strategy update steps.

We report the long-run population-average frequency of cooperation in the evolving population and the long-run average strategy of the population. We start our simulation with a population where all individuals adopt an ALLD-type strategy (0,0.01,0.01,0.01,0.01,0,0). Average cooperation in the population is calculated as the long-run frequency of the action “C” across all repeated games possible under random matching.

## Supplementary Material

pgag005_Supplementary_Data

## Data Availability

There are no data underlying this work.
