# Supplementary material for: From simultaneous to leader–follower play in direct reciprocity
Source: PNAS Nexus. 2026 Jan 13;5(2):pgag005. doi: 10.1093/pnasnexus/pgag005 (PMC12880187; doi:10.1093/pnasnexus/pgag005)
Supplement: pgag005_Supplementary_Data [file pgag005_supplementary_data.pdf]

Supporting Information:
From simultaneous to leader-follower play in direct reciprocity

Contents

Model **2**

The basic repeated game . . . 2

Leader-follower stability . . . 3

Failure of leader-follower stability . . . 5

Results **8**

Memory-1 equilibria . . . 8

What happens as $\delta \to 1$? . . . 9

Dilemma games . . . 10

Equalizer equilibria . . . 11

Equilibria in memory-n . . . 12

Methods **13**

Memory reduction . . . 13

Memory-1 Markov chains and payoffs . . . 14

Equilibrium types . . . 15

Derivation of main results . . . 16

Equalizers . . . 19

Appendix: Mathematical tools **21**

Markov chains . . . 21

Markov decision processes . . . 21

Model

The basic repeated game

We study an infinitely repeated game between two players. In each round, each player chooses from the same finite set of actions. Unless otherwise specified, we assume the players choose between just two actions, which we call C and D. The game is symmetric. The payoff to the row player is specified by a fixed payoff matrix

	C	D
C	a	b
D	c	d

(1)

Occasionally we write the payoff matrix (11) as (a, b, c, d) for brevity. Occasionally we may refer to the actions C and D as 'cooperation' and 'defection,' respectively. However, this is just a convention. We make no assumptions about the interpretations of the actions, or the payoffs $a, b, c, d \in \mathbb{R}$.

The outcome of each round is an action profile $\mathbf{a} \in \{\text{CC, CD, DC, DD}\}$. Here CD means that the focal player played C and the opponent played D in that round. The history of the game is given by a sequence of round outcomes. For example, at time $t+1$ there is a game history $\mathbf{h} = (\mathbf{a}_1, \mathbf{a}_2, \ldots, \mathbf{a}_t)$ which describes the actions taken in the previous t rounds. The set of all possible game histories at all finite times is

$$\mathbf{H} = \{(\mathbf{a}_1, \mathbf{a}_2, \ldots, \mathbf{a}_t) \ : \ \mathbf{a}_j \in \{\text{CC, CD, DC, DD}\}, \ t \in \{0, 1, 2, \ldots\}\} \tag{2}$$

A **strategy** for the repeated game is a map $\sigma : \mathbf{H} \to [0, 1]$. The interpretation is as follows. Suppose $\mathbf{h} = (\mathbf{a}_1, \mathbf{a}_2, \ldots, \mathbf{a}_t)$ is the history of the game up until the current round. Then the strategy σ plays C in the current round with probability $\sigma(\mathbf{h})$. If $\sigma(\mathbf{h}) \in \{0, 1\}$ for all histories $\mathbf{h}$, then the strategy is deterministic. We write Σ to denote the set of all strategies σ.

Suppose that player 1 uses a strategy σ_1 and player 2 uses a strategy σ_2. Let $\pi_\delta(\sigma_1, \sigma_2)$ denote the discounted payoff to player 1, defined as

$$\pi_\delta(\sigma_1, \sigma_2) := (1 - \delta) \sum_{t=0}^{\infty} \delta^t \pi(t) \tag{3}$$

Here $\pi(t)$ is the expected payoff that player 1 receives in round t of the game. This depends on σ_1 and σ_2. The fixed value $\delta \in (0, 1)$ is called the discount factor of the repeated game.

A profile of strategies (σ_1, σ_2) constitutes a **Nash equilibrium** if the following inequalities hold for every $\sigma_1', \sigma_2' \in \Sigma$:

$$\begin{aligned} \pi_\delta(\sigma_1, \sigma_2) &\geqslant \pi_\delta(\sigma_1', \sigma_2) \\ \pi_\delta(\sigma_2, \sigma_1) &\geqslant \pi_\delta(\sigma_2', \sigma_1) \end{aligned} \tag{4}$$

A Nash equilibrium profile (σ_1, σ_2) is **deterministic along the equilibrium path** if — when σ_1 plays against σ_2 — each move by each player is selected with no randomization. Many Nash equilibria of interest have this property, even when the strategies themselves are not completely deterministic. Such strategies may still randomize between actions if the equilibrium is disturbed.

A **memory-**1 **strategy** for the repeated game always chooses the next move based on the outcome of the previous round only. Formally, a memory-1 strategy $\mathbf{p} = (p_0; p_{\mathrm{CC}}, p_{\mathrm{CD}}, p_{\mathrm{DC}}, p_{\mathrm{DD}})$ is specified by five probabilities: p_0 is the probability for playing C in the first round, and p_{XY} is the probability for playing C next if the focal player's own last move was X and the opponent's last move was Y. For conciseness we usually write $\mathbf{p} = (p_0; p_1, p_2, p_3, p_4)$. We denote the space of memory-1 strategies by $\mathbf{Mem}^1$.

A **memory-**n **strategy** for the repeated game always chooses the next move based on the outcome of the previous n rounds only. (In the first n rounds, the strategy has some arbitrary fixed specification for how to move as a function of the game history.) We denote the space of memory-n strategies by $\mathbf{Mem}^n$.

Leader-follower stability

In the standard repeated game, both players choose moves simultaneously and without knowing the other's concurrent move. A strategy is a map $\sigma : \mathbf{H} \to [0, 1]$.

In the main text we have introduced a modification to the game. We suppose that a given player is forced to commit to a move just before the co-player, who can observe this move. Equivalently, we suppose that the co-player can perfectly predict the focal player's concurrent move as the co-player chooses a move. The effect is that one player is the "leader" and one player is the "follower" in each round. Rather than explicitly deal with two different game structures, we find it convenient to reflect the difference in the strategies themselves. Thus, we say a **follower-type strategy** is a map

$$\sigma : \mathbf{H} \times \{\mathrm{C}, \mathrm{D}\} \to [0, 1] \tag{5}$$

The interpretation is as follows. By construction, a follower-type strategy forces the opponent into the role of a leader. If the history of the game up to the present time is described by $\mathbf{h} \in \mathbf{H}$; and the move of the leader in the current round is $X \in \{\mathrm{C}, \mathrm{D}\}$; then the follower plays C with probability $\sigma(\mathbf{h}, X)$. Here X is the observed move of the leader in the current round.

Because the leader moves first and the follower moves second in each round, the leader must use an ordinary strategy, which looks at the previous rounds only and decides how to move. Possibly the follower may also decide to use an ordinary strategy. After all, the space of all follower-type strategies contains, as a subset, the space of all ordinary strategies (for which there is no X-dependence). If the follower does so, then the leader-follower structure is irrelevant and the game is effectively simultaneous.

In general it does not make sense for two players to use follower-type strategies against each other. In that case, player 1's move would be based on a prediction or observation of player 2's move, which in turn is based on a prediction or observation of player 1's move. This generates a causal loop which, in general, admits no consistent resolution.

The simplest follower-type strategies are the memory-0 follower-type strategies, denoted by $\mathbf{FMem}^0$.

A **memory-0 follower-type strategy** chooses a move based only on the move of the leader in the current round. It can be written as a pair $\mathbf{p} = (\lambda_1, \lambda_2)$, where λ_1 is the probability of playing C when the co-player plays C concurrently, and λ_2 is the probability of playing C when the co-player plays D concurrently.

There are four such deterministic strategies:

1. ALLD $= (0, 0)$, always plays D. This behavior is also captured by the memory-1 strategy $(0; 0, 0, 0, 0)$ with the same name.

2. ALLC $= (1, 1)$, always plays C. This behavior is also captured by the memory-1 strategy $(1; 1, 1, 1, 1)$ with the same name.

3. COPY $= (1, 0)$, always plays the same action as the leader (note, in the *same* round). COPY is not to be confused with the well-known memory-1 strategy TFT, which plays the action that the opponent played in the *previous* round.

4. ACOPY $= (0, 1)$, always plays the opposite action as the leader. ACOPY, or anti-COPY, is not to be confused with the memory-1 strategy anti-TFT.

More generally, a **memory-n follower-type strategy** chooses a move based on the outcome of the previous n rounds, together with the leader's move in the current round. (In the first n rounds, the strategy has some arbitrary fixed specification for how to move as a function of the game history and the leader's move.) We denote the space of memory-n follower-type strategies by $\mathbf{FMem}^n$.

If σ_1 is a follower-type strategy, and σ_2 is an ordinary strategy, then we still write $\pi_\delta(\sigma_1, \sigma_2)$ for the discounted payoff to player 1. We say that a profile of strategies $(\sigma_1, \sigma_2) \in \Sigma \times \Sigma$ constitutes an **leader-follower stable Nash equilibrium**, or **LF-stable Nash equilibrium**, if the following inequalities hold for every $\sigma_1', \sigma_2' \in \Sigma_{\mathrm{F}}$:

$$\begin{aligned} \pi_\delta(\sigma_1, \sigma_2) &\geqslant \pi_\delta(\sigma_1', \sigma_2) \\ \pi_\delta(\sigma_2, \sigma_1) &\geqslant \pi_\delta(\sigma_2', \sigma_1) \end{aligned} \tag{6}$$

The condition for an LF-stable Nash equilibrium is just like the condition for a Nash equilibrium, except that σ_1', σ_2' range over the set Σ_{F} of follower-type strategies rather than the smaller subset Σ of ordinary strategies. Another way of saying this is that an LF-stable Nash equilibrium is a Nash equilibrium for the simultaneous game, which remains Nash in the associated leader-follower game, when one player is designated as the leader.

Note that if the strategies in a Nash equilibrium are completely deterministic, then the equilibrium is automatically LF-stable. Follower-type opponents have no advantage over ordinary opponents, because the action of a deterministic strategy is always determined by the previous rounds only. Therefore, this information can be known and used by any opponent with sufficient memory, even if the opponent must move simultaneously.

However, many strategies of interest in evolutionary game theory have some stochastic components. For example, the well-known strategy generous tit-for-tat (GTFT) plays C with some probability $0 <$

$q < 1$ if the co-player played D in the previous round. When a Nash equilibrium consists of strategies with stochastic components, it is unclear *a priori* whether or not the equilibrium is LF-stable. This is true even if the equilibrium is deterministic at equilibrium; in principle, a predictive strategy could deliberately upset the equilibrium in order to exploit the stochasticity of subsequent actions, somehow gaining a net profit. We give detailed examples below, adding rigorous arguments to the brief descriptions in the main text:

Failure of leader-follower stability

Nash equilibria can fail to be leader-follower stable when the strategies involved have stochastic components. We give three examples. In these examples, we give exact specifications of payoff matrices and probabilities, but we observe that *the qualitative phenomena remain unchanged under small perturbations*.

Example 1: Consider a repeated Snowdrift game with payoff matrix

	C	D
C	3	$\frac{5}{2}$
D	5	1

(7)

Consider a memory-2 strategy σ, which plays C if neither player has played D in the previous two rounds, and otherwise plays C with probability $3/7$.

We claim that the strategy profile (σ, σ) is a Nash equilibrium when δ is large. When both players use σ, they always play C and each get total discounted payoff 3. If one player deviates and plays D in a certain round, then one can calculate that it takes at least 7.7 rounds on average to return to a state in which neither player has played D in the previous two rounds. This is true regardless of the deviating player's moves (the best case scenario is that the deviating player plays C in every subsequent round). In each these intermediate rounds, the deviating player expects to earn approximately 2.7 on average, regardless of their chosen action. So the deviating player would lose on average at least $(3-2.7) \cdot 7.7 = 2.31$ in total cumulative payoff during this period, while gaining only $+2$ for the deviation, in the first round of playing D. (To make this argument completely rigorous, one could use Doob's optional stopping theorem (1).) Therefore, for a discount factor δ close to 1, the deviation from always playing C cannot be profitable.

On the other hand, this Nash equilibrium is not LF-stable. There is a follower-type strategy which, after two consecutive rounds of CC, plays D; and subsequently, simply plays the opposite of the opponent's move. It is simple to calculate the expected per-round payoff this strategy earns in the long run (that is, the payoff as $\delta \to 1$): $5 \cdot (3/7) + (5/2) \cdot (4/7) \approx 3.57$. The expected total discounted payoff for δ close to 1 will be similar. Since $3.57 > 3$, deviation to a follower-type strategy is profitable.

Example 2: A repeated donation game with cost 2 and benefit 3, has payoff matrix

$$\begin{array}{c|cc} & \text{C} & \text{D} \\ \hline \text{C} & 1 & -2 \\ \text{D} & 3 & 0 \end{array} \tag{8}$$

To define a memory-3 strategy, we use the following convention. The outcome of three consecutive rounds will be represented as a sequence in chronological order, separated by '|'. For example, a memory-3 history could be CC|CD|DD. Now consider a memory-3 strategy σ defined as follows:

1. If neither player has played D during the previous 3 rounds, the strategy σ plays C. (This implies, for instance, that the first move is C.)
2. If the previous three rounds are described by an element of the following set, the strategy σ plays C:
$$\{\text{CC|CC|CC, DD|DD|CC, DD|CC|CD, CC|CD|CD}\} \tag{9}$$
3. After a memory-3 history DD|DD|DD, the strategy σ plays C with probability $1/2$.
4. Otherwise, the strategy plays D.

Against a copy of itself, this strategy always plays C and earns total discounted payoff 1. We claim this is a Nash equilibrium.

A best reply σ' to σ can be found amongst the deterministic memory-3 strategies (2). Suppose σ is not a best reply to itself. Then we can assume σ' is the first to play D, at some point in the first four rounds. This causes σ to play D in the next round and until three consecutive rounds of DD occur. Until DD|DD|DD occurs, σ' can earn at most 0 in each round, which is less than the equilibrium per-round payoff. But σ' must earn *more* per round, in the long run, than the equilibrium per-round payoff (assuming δ close to 1). So, whenever σ is locked into playing D until DD|DD|DD, we can assume σ' does not waste any rounds but instead also plays D three times in a row, immediately achieving DD|DD|DD. After DD|DD|DD, σ plays C with probability $1/2$.

If σ' plays D deterministically after DD|DD|DD, then the game between the two players will become consecutive sequences of DD interspersed occasionally with a single round of CD. Each sequence of DD is length at least 3, so this cannot be profitable (assuming δ close to 1).

So σ' plays C deterministically in the round after DD|DD|DD. There are two possibilities, each with probability $1/2$, for this round:

Case 1: σ plays D. Then σ will again play D until DD|DD|DD, after which we restart at the two cases.

Case 2: σ plays C. Then the new history is DD|DD|CC. Now, we claim σ' must play D three times in a row, which will result in CD|CD|CD. Of course, after this σ will play D until DD|DD|DD again. But this same undesirable epilogue only happens more quickly — and with less profit — with any other deterministic behavior from σ'.

We have already specified enough behavior to determine how much σ' earns. After equilibrium is disturbed and DD|DD|DD is achieved, the game is described by a Markov chain on the set of ten states

DD|DD|DD, DD|DD|DC, DD|DC|DD, DC|DD|DD, DD|DD|CC, DD|CC|CD, CC|CD|CD, CD|CD|CD, CD|CD|DD, CD|DD|DD. The transition matrix is clear from the above description of σ and σ'. The long-run per-round average payoff to σ' can be calculated by analyzing the stationary distribution of the Markov chain. It is $8/11$, which is less than equilibrium payoff 1.

On the other hand, there is a follower-type strategy σ'' which behaves exactly the same as σ', except that when the previous three rounds are DD|DD|DD, the strategy σ'' plays C if and only if σ plays C concurrently. By the same methods, the long-run per-round average payoff to σ'' is $5/4$, which is greater than the equilibrium payoff 1.

When δ is close to 1, the expected total discounted payoff is close to the long-run per-round average payoff. So for some such δ we have shown that the strategy profile (σ, σ) is a Nash equilibrium which is not LF-stable.

Example 3: Consider a three-action game with payoff matrix

	C	D	E
C	10	-20	-1
D	-20	10	-1
E	-1	-1	0

(10)

There is a memory-1 strategy σ which plays E after the outcome EE, and otherwise plays C or D, each with probability $1/2$. The strategy profile (σ, σ) is a Nash equilibrium in which both players always play E. If one player deviates to C or D, then that player earns an expected payoff of ≤ -1 in every subsequent round, regardless of the action which the deviating player takes. So this deviation is not profitable, even in the short run.

On the other hand, there is a follower-type strategy which plays D in the first round and subsequently mimics the opponent's action in each round. If one of the two players in the equilibrium (σ, σ) deviates to this strategy, they will earn 10 in every round after the first, so the deviation is profitable.

Remark. The reader may want to see examples similar to those in Examples 2-3, but in which the Nash equilibrium strategy profile (σ, σ) is guaranteed to eventually return to the stable equilibrium behavior after an error. That can be achieved in Example 2 by introducing a very small probability to play C after DD|CC|CC, and in Example 3 by introducing a very small probability to play E after any action profile. For fixed $\delta < 1$, the expected total payoffs are continuous functions of the strategies. So it is easy to see that our conclusions still hold after these modifications.

Results

The results below refer to the repeated 2×2 symmetric game with discount factor $\delta \in (0, 1)$ and payoff matrix

$$\begin{array}{c|cc} & \text{C} & \text{D} \\ \hline \text{C} & a & b \\ \text{D} & c & d \end{array} \tag{11}$$

We remind the reader that $a, b, c, d \in \mathbb{R}$ are arbitrary.

Memory-1 equilibria

A **symmetric memory-1 Nash equilibrium** is a Nash equilibrium consisting of two memory-1 strategies $\mathbf{p} = (p_0; p_1, p_2, p_3, p_4)$ and $\mathbf{q} = (q_0; q_1, q_2, q_3, q_4)$ such that $p_i = q_i$ for $i = 1, 2, 3, 4$ (not necessarily $i = 0$).

We present results on symmetric memory-1 Nash equilibrium profiles (σ_1, σ_2) which are deterministic along the equilibrium path.

Proposition 1. *There are five types of symmetric memory-1 Nash equilibria which are deterministic along the equilibrium path: mutual cooperation, mutual defection, trans-alternation, cis-alternation, and CD-repetition. For each, we pick a conventional choice of starting moves and derive analytical conditions for Nash equilibrium below:*

1. *(Cooperation) The strategy profile* $(1; 1, p_2, p_3, p_4), (1; 1, p_2, p_3, p_4)$ *is a Nash equilibrium if and only if*

$$(1 - p_2)(a - d) + p_4(a - c) \geqslant \left(\frac{1 - \delta}{\delta}\right)(c - a)$$
$$(1 - p_2)(a - b) + p_3(a - c) \geqslant \left(\frac{1 - \delta}{\delta}\right)(c - a)$$

2. *(Defection) The strategy profile* $(0; p_1, p_2, p_3, 0), (0; p_1, p_2, p_3, 0)$ *is a Nash equilibrium if and only if*

$$p_3(a - d) + p_1(d - b) \leqslant \left(\frac{1}{\delta}\right)(d - b)$$
$$p_3(c - d) + p_2(d - b) \leqslant \left(\frac{1}{\delta}\right)(d - b)$$

3. *(trans-Alternation) The strategy profile* $(1; p_1, 0, 1, p_4), (0; p_1, 0, 1, p_4)$ *is a Nash equilibrium if and*

only if

$$p_1(c-b) \leqslant \left(\frac{1+\delta}{\delta}\right)(c-a)$$
$$(1-p_4)(c-b) \geqslant \left(\frac{1+\delta}{\delta}\right)(d-b)$$

4. *(cis-Alternation) The strategy profile* $(1;0,p_2,p_3,1),(1;0,p_2,p_3,1)$ *is a Nash equilibrium if and only if*

$$p_2(a-d) \leqslant \left(\frac{1+\delta}{\delta}\right)(a-c)$$
$$(1-p_3)(a-d) \geqslant \left(\frac{1+\delta}{\delta}\right)(b-d)$$

5. *(CD-Repetition) The strategy profile* $(1;p_1,1,0,p_4),(0;p_1,1,0,p_4)$ *is a Nash equilibrium if and only if*

$$(1-p_1)(b-c) \leqslant \left(\frac{1-\delta}{\delta}\right)(c-a)$$
$$p_4(c-b) \leqslant \left(\frac{1-\delta}{\delta}\right)(b-d)$$
$$(1-p_1)(c-d)+p_4(c-a) \geqslant \left(\frac{1-\delta}{\delta}\right)(a-c)$$
$$p_1(d-b)+p_4(b-a) \geqslant \left(\frac{1}{\delta}\right)(d-b)$$

Proposition 2. *Every Nash equilibrium listed above is LF-stable.*

Proposition 2 describes rather special case: memory-1 strategies in repeated games with two-actions. In fact, the analogue of Proposition 2 does not hold for repeated games with more than two actions, or for strategies with higher memory. We have shown this already in the subsection Failure of leader-follower stability.

What happens as $\delta \to 1$?

If the inequalities in Proposition 1 are strict when $\delta = 1$, then they hold for all large δ, that is, for all $\delta < 1$ in some neighborhood of 1. This can only happen for the first four types. We have the following proposition:

Proposition 3.

1. *(Cooperation) The strategy profile* $(1;1,p_2,p_3,p_4),(1;1,p_2,p_3,p_4)$ *is a Nash equilibrium for all*

large δ if

$$(1-p_2)(a-d)+p_4(a-c)>0$$
$$(1-p_2)(a-b)+p_3(a-c)>0$$

2. *(Defection) The strategy profile* $(0;p_1,p_2,p_3,0),(0;p_1,p_2,p_3,0)$ *is a Nash equilibrium for all large δ if*

$$p_3(a-d)+(1-p_1)(b-d)<0$$
$$p_3(c-d)+(1-p_2)(b-d)<0$$

3. *(trans-Alternation) The strategy profile* $(1;p_1,0,1,p_4),(0;p_1,0,1,p_4)$ *is a Nash equilibrium for all large δ if*

$$p_1(c-b)<2(c-a)$$
$$(1-p_4)(c-b)>2(d-b)$$

4. *(cis-Alternation) The strategy profile* $(1;0,p_2,p_3,1),(1;0,p_2,p_3,1)$ *is a Nash equilibrium for all large δ if*

$$p_2(a-d)<2(a-c)$$
$$(1-p_3)(a-d)>2(b-d)$$

The conditions above are sufficient conditions, unlike the ones in Proposition 1, which are necessary and sufficient. Necessary and sufficient conditions for a strategy profile to be a Nash equilibrium for all large δ can be easily derived from Proposition 1 by including some extra edge cases.

Dilemma games

The results in the previous subsection can be used to derive interesting consequences for a given payoff matrix (a,b,c,d). Here we assume $a-d>0$, and normalize the payoff matrix to the following form by subtracting a constant from each entry and multiplying by a positive constant (3):

$$(1,u,1+v,0):=\frac{1}{(a-d)}\Big[(a,b,c,d)-(d,d,d,d)\Big] \tag{12}$$

The resulting space of payoff matrices can be parameterized as a plane with coordinates u,v. The four quadrants of this plane correspond to payoff matrices with different equilibrium properties for the corresponding *one-shot* game: respectively, Quadrant I ($u,v>0$) corresponds to Snowdrift games, in which the action profiles CD and DC are Nash equilibria; Quadrant II ($u>0,v>0$) corresponds to Prisoner's

dilemma games, in which the action profile DD is the unique Nash equilibrium; Quadrant III ($u, v < 0$) corresponds to Stag-hunt games, in which the two action profiles CC and DD are Nash equilibria; and Quadrant IV ($u > 0, v < 0$) corresponds to Harmony games, in which the action profile CC is the unique Nash equilibrium. We could call all of these cooperation games or dilemma games, in a generalized sense.

Proposition 4. *For the repeated game with payoff matrix* $(1, u, 1+v, 0)$*:*

1. *(Cooperation) There is a Nash equilibrium profile of the form* $(1; 1, p_1, p_2, p_3, p_4)$*,* $(1; 1, p_2, p_3, p_4)$ *if and only if*
$$v \leqslant 0 \;\; \textit{or} \;\; v(1-\delta) \leqslant \delta \min(1, 1-u) \tag{13}$$
2. *(Defection) There is a Nash equilibrium profile of the form* $(0; p_1, p_2, p_3, 0)$*,* $(0; p_1, p_2, p_3, 0)$ *if and only if* $u \leqslant 0$*.*
3. *(trans-Alternation) There is a Nash equilibrium profile of the form* $(1; p_1, 0, 1, p_4)$*,* $(0; p_1, 0, 1, p_4)$ *if and only if*
$$\begin{aligned} &v \geqslant 0 \;\; \textit{and} \;\; -\delta(1+v) \leqslant u \leqslant (1+v), \;\; \textit{or} \\ &u \geqslant 0 \;\; \textit{and} \;\; \delta(1-u) \leqslant v \leqslant -(1-u) \end{aligned} \tag{14}$$
4. *(cis-Alternation) There is a Nash equilibrium profile of the form* $(1; 0, p_2, p_3, 1)$*,* $(1; 0, p_2, p_3, 1)$ *if and only if*
$$v \leqslant 0 \;\; \textit{and} \;\; (1+\delta)u \leqslant \delta \tag{15}$$
5. *(CD-Repetition) There is a Nash equilibrium profile of the form* $(1; p_1, 1, 0, p_4)$*,* $(0; p_1, 1, 0, p_4)$ *if and only if* $u \geqslant 0$ *and* $v \geqslant 0$*.*

Equalizer equilibria

A memory-1 strategy $\mathbf{p} = (-; p_1, p_2, p_3, p_4)$ is called an **equalizer**(4; 5) if, for any given p_0, the payoff to the opponent of $\mathbf{p}$ is independent of the opponent's strategy. The equalizer strategies are the solutions of the system
$$\frac{c-d}{1-\delta(p_2-p_4)} = \frac{a-b}{1-\delta(p_1-p_3)} = \frac{a-d}{1-\delta(p_1-p_4)} \tag{16}$$
(See Methods, as well as (5).)

Proposition 5. *Every equalizer strategy is also a* ***leader-follower equalizer****: the payoff to the opponent is the same even if the opponent is allowed to use follower-type strategies.*

This is noteworthy, as equalizer strategies form Nash equilibria which in general are mixing, i.e. not deterministic along the equilibrium path.

Equilibria in memory-n

Recall that $\mathbf{Mem}^n$ is the space of memory-n strategies, and $\mathbf{FMem}^n$ is the space of memory-n predictive strategies. The following statement is well known from standard theory (2):

Proposition 6. *A profile of memory-n strategies $(\mathbf{p}, \mathbf{q})$ constitutes a Nash equilibrium if and only if the following inequalities hold for all deterministic strategies $\mathbf{p}', \mathbf{q}' \in \mathbf{Mem}^n$:*

$$\begin{aligned} \pi_\delta(\mathbf{p}, \mathbf{q}) &\geqslant \pi_\delta(\mathbf{p}', \mathbf{q}) \\ \pi_\delta(\mathbf{q}, \mathbf{p}) &\geqslant \pi_\delta(\mathbf{q}', \mathbf{p}) \end{aligned} \tag{17}$$

A new and potentially useful statement is the following:

Proposition 7. *A profile of memory-n strategies $(\mathbf{p}, \mathbf{q})$ constitutes an LF-stable Nash equilibrium if and only if the following inequalities hold for all deterministic strategies $\mathbf{p}', \mathbf{q}' \in \mathbf{FMem}^{n-1}$:*

$$\begin{aligned} \pi_\delta(\mathbf{p}, \mathbf{q}) &\geqslant \pi_\delta(\mathbf{p}', \mathbf{q}) \\ \pi_\delta(\mathbf{q}, \mathbf{p}) &\geqslant \pi_\delta(\mathbf{q}', \mathbf{p}) \end{aligned} \tag{18}$$

In particular, Proposition 7 gives an interesting sufficient condition for a profile of memory-n strategies to be a Nash equilibrium. The number of deterministic strategies in $\mathbf{FMem}^{n-1}$ is substantially smaller than the number of deterministic strategies in $\mathbf{Mem}^n$, which is useful for computation. In our definition, we allowed the behavior of a strategy in the initial rounds to be arbitrary. This means the number of deterministic strategies in $\mathbf{Mem}^n$ is $\prod_{i=0}^{n} 2^{4^i}$ and the number of deterministic strategies in $\mathbf{FMem}^{n-1}$ is $\prod_{i=0}^{n-1} 2^{2\cdot 4^i}$. The ratio between these is $2^{(2\cdot 4^n+1)/3}$. It is much easier to verify LF-stable Nash equilibria than to verify Nash equilibria, at least by naïve brute force computation.

Methods

In this section we present proofs of the main results.

Memory reduction

The condition for a strategy profile to be a Nash equilibrium or to be LF-stable depends on the payoffs achieved by all possible opponents. In this subsection we show that it is sufficient to consider only a small set of opponents with bounded memory.

Suppose player 2 uses a fixed memory-1 strategy $\mathbf{q} = (q_0; q_1, q_2, q_3, q_4)$.

From the perspective of player 1, player 2 can be taken for granted as a part of a stochastic environment. Then the repeated game is nothing but a Markov decision process (or one-player stochastic game) on the set of round outcomes $S = \{\text{CC, CD, DC, DD}\}$. Formally, the action set is $A = \{\text{C, D}\}$. The transition probability is given by

$$p_{\text{C}}(i,j) = \begin{pmatrix} q_1 & (1-q_1) & 0 & 0 \\ q_3 & (1-q_3) & 0 & 0 \\ q_2 & (1-q_2) & 0 & 0 \\ q_4 & (1-q_4) & 0 & 0 \end{pmatrix}, \quad p_{\text{D}}(i,j) = \begin{pmatrix} 0 & 0 & q_1 & (1-q_1) \\ 0 & 0 & q_3 & (1-q_3) \\ 0 & 0 & q_2 & (1-q_2) \\ 0 & 0 & q_4 & (1-q_4) \end{pmatrix} \tag{19}$$

The reward functions $r_{\text{C}}(i)$ and $r_{\text{D}}(i)$ are both given by (a, b, c, d). For example, $r_{\text{C}}(\text{CD})$ is the expected reward which player 1 receives in a round for which the outcome is CD, and assuming that player 1 plays C next. Of course, the new action — that is, the subscript C or D — does not matter for the payoff of the completed round. It only affects the transition probability to the outcome of the next round. That is why $r_{\text{C}}(i) = r_{\text{D}}(i)$. We refer to this Markov decision process as the **associated memory-1 MDP** for the game and the strategy $\mathbf{q}$.

By Proposition 10 in the Appendix, we can say that a best reply to $\mathbf{q}$ can be found in in the space of memory-1 strategies (i.e. in the space of stationary strategies for the associated memory-1 MDP). In other words, the space of memory-1 strategies is closed under the operation of taking best replies. An immediate corollary is the following:

Proposition 8. *A profile of memory-1 strategies $(\mathbf{p}, \mathbf{q})$ constitutes a Nash equilibrium if and only if the following inequalities hold for all deterministic strategies $\mathbf{p}', \mathbf{q}' \in \mathbf{Mem}^1$:*

$$\pi_\delta(\mathbf{p}, \mathbf{q}) \geqslant \pi_\delta(\mathbf{p}', \mathbf{q})$$
$$\pi_\delta(\mathbf{q}, \mathbf{p}) \geqslant \pi_\delta(\mathbf{q}', \mathbf{p})$$

Now suppose that player 1 is the follower in the repeated leader-follower version of the game, and always knows player 2's move in the current round. Now, from the perspective of such a player, the game can be described by a Markov decision process on the set of states $S = \{\text{C, D}\}$. The action set, as before,

is $A = \{\text{C}, \text{D}\}$. The current state describes player 2's move in the current round, which player 1 can observe.

For this MDP, the transition probability is given by

$$p_C(i,j) = \begin{pmatrix} q_1 & 1-q_1 \\ q_2 & 1-q_2 \end{pmatrix}, \quad p_D(i,j) = \begin{pmatrix} q_3 & 1-q_3 \\ q_4 & 1-q_4 \end{pmatrix} \tag{20}$$

The reward functions are $r_C(i) = (a, b)$ and $r_D(i) = (c, d)$. These rewards capture the payoff from the current round (i.e. the round for which player 2's current move has been observed). We refer to this Markov decision process as the **associated leader-follower MDP** for the game and the strategy $\mathbf{q}$.

Now, by Proposition 10 in the Appendix, we can say that a best reply to $\mathbf{p}$, among the space of all follower-type strategies, can be found among the space of memory-0 follower-type strategies (i.e. the space of stationary strategies for the MDP). An immediate corollary is the following:

Proposition 9. *A profile of memory-*1 *strategies* $(\mathbf{p}, \mathbf{q})$ *constitutes a leader-follower stable Nash equilibrium if and only if the following inequalities hold for all deterministic strategies* $\mathbf{p}', \mathbf{q}' \in \mathbf{FMem}^0$*:*

$$\begin{aligned} \pi_\delta(\mathbf{p}, \mathbf{q}) &\geqslant \pi_\delta(\mathbf{p}', \mathbf{q}) \\ \pi_\delta(\mathbf{q}, \mathbf{p}) &\geqslant \pi_\delta(\mathbf{q}', \mathbf{p}) \end{aligned} \tag{21}$$

Propositions 8-9 accomplish a huge reduction in memory which make it feasible to analytically characterize memory-1 Nash equilibria and test them for LF-stability. These propositions effectively say that deviations to higher-memory strategies need not be considered.

The techniques used to prove Propositions 8-9 can also be applied to higher memory strategies. By defining an appropriate Markov decision process in each case, it is possible to establish Propositions 6-7. We omit formal proofs.

Memory-1 Markov chains and payoffs

If player 1 uses a memory-1 strategy $\mathbf{p}$ and player 2 uses a memory-1 strategy $\mathbf{q}$, then the repeated game is described by a Markov chain $M(\mathbf{p}, \mathbf{q})$ with state space $\{\text{CC}, \text{CD}, \text{DC}, \text{DD}\}$. The state CD, for example, refers to a round in which player 1 played C and player 2 played D. The transition matrix is

$$\mathbf{M} = \begin{pmatrix} p_1 q_1 & p_1(1-q_1) & (1-p_1)q_1 & (1-p_1)(1-q_1) \\ p_2 q_3 & p_2(1-q_3) & (1-p_2)q_3 & (1-p_2)(1-q_3) \\ p_3 q_2 & p_3(1-q_2) & (1-p_3)q_2 & (1-p_3)(1-q_2) \\ p_4 q_4 & p_4(1-q_4) & (1-p_4)q_4 & (1-p_4)(1-q_4) \end{pmatrix} \tag{22}$$

Recall that a memory-0 predictive strategy $\mathbf{p} = (\lambda_1, \lambda_2)$ gives two probabilities for cooperating λ_1, λ_2 based on whether the opponent's concurrent move is C or D, respectively. The space $\mathbf{FMem}^0$ is the unit square $[0, 1]^2$.

When player 1 uses a memory-0 follower-type strategy $\mathbf{p} = (\lambda_1, \lambda_2)$ and player 2 uses a memory-1 strategy $\mathbf{q} = (q_1, q_2, q_3, q_4)$, the gameplay is described by a Markov chain $M(\mathbf{p}, \mathbf{q})$ with state space $\{\mathrm{CC}, \mathrm{CD}, \mathrm{DC}, \mathrm{DD}\}$ and transition matrix

$$\mathbf{M} = \begin{pmatrix} \lambda_1 q_1 & \lambda_2(1-q_1) & (1-\lambda_1)q_1 & (1-\lambda_2)(1-q_1) \\ \lambda_1 q_3 & \lambda_2(1-q_3) & (1-\lambda_1)q_3 & (1-\lambda_2)(1-q_3) \\ \lambda_1 q_2 & \lambda_2(1-q_2) & (1-\lambda_1)q_2 & (1-\lambda_2)(1-q_2) \\ \lambda_1 q_4 & \lambda_2(1-q_4) & (1-\lambda_1)q_4 & (1-\lambda_2)(1-q_4) \end{pmatrix} \tag{23}$$

In any case, given a Markov chain for a repeated game, there is a simple way of calculating the discounted payoff to both players. Suppose the Markov chain has transition matrix $\mathbf{M}$. The probabilities of the outcomes CC, CD, DC, DD in the first round are given by some vector $\mathbf{v}$. For two memory-1 strategies, we have

$$\mathbf{v} = (p_0 q_0, p_0(1-q_0), (1-p_0)q_0, (1-p_0)(1-q_0)) \tag{24}$$

For a memory-0 follower-type strategy $\mathbf{p} = (\lambda_1, \lambda_2)$ and a memory-1 strategy $\mathbf{q} = (q_0; q_1, q_2, q_3, q_4)$, we have

$$\mathbf{v} = (\lambda_1 q_0, \lambda_2(1-q_0), (1-\lambda_1)q_0, (1-\lambda_2)(1-q_0)) \tag{25}$$

In either case, the expected payoff to player 1 from the first round is equal to the dot product $\mathbf{v} \cdot (a, b, c, d)$. The probabilities of the outcomes CC, CD, DC, DD in round t are given by the vector $\mathbf{v}\mathbf{M}^{t-1}$. So the discounted payoff to player 1 is, by definition,

$$\begin{aligned} \pi_\delta(\mathbf{p}, \mathbf{q}) &= (1-\delta) \sum_{i=0}^{\infty} \delta^i (\mathbf{v}\mathbf{M}^i) \cdot (a, b, c, d) \\ &= (1-\delta)\mathbf{v}(\mathbf{I} - \delta\mathbf{M})^{-1} \cdot (a, b, c, d) \\ &= \mathbf{w}_{\mathbf{pq}} \cdot (a, b, c, d) \end{aligned} \tag{26}$$

Here $\mathbf{I}$ is the identity matrix, and we use the shorthand

$$\mathbf{w}_{\mathbf{pq}} := (1-\delta)\mathbf{v}(\mathbf{I} - \delta\mathbf{M})^{-1} \tag{27}$$

To calculate the payoff for player 2, note that one can simply switch b and c:

$$\pi_\delta(\mathbf{q}, \mathbf{p}) = \mathbf{w}_{\mathbf{pq}} \cdot (a, c, b, d) \tag{28}$$

Equilibrium types

Consider a symmetric memory-1 Nash equilibrium, that is, a Nash equilibrium strategy profile $(\mathbf{p}, \mathbf{q})$ of memory-1 strategies with $p_i = q_i$ for $i = 1, 2, 3, 4$. Suppose this Nash equilibrium is deterministic along the equilibrium path. We examine what this implies for the variables p_i.

A deterministic transition CC→CD is impossible because it implies $p_1 = 1 \neq q_1 = 0$. Likewise,

a deterministic transition CC→DC is impossible. So there are three cases: either (i) CC never occurs at equilibrium, or (ii) CC is an absorbing state, or else (iii) CC transitions to DD deterministically. By symmetry, each of these has three subcases: (a) DD never occurs at equilibrium, or (b) DD is an absorbing state, or else (c) DD transitions to CC deterministically.

In case (ii), we have $p_1 = q_1 = 1$. Since CC is an absorbing state played at equilibrium, the equilibrium reaches mutual **cooperation**. In case (b), we have $p_4 = q_4 = 0$. Since DD is an absorbing state played at equilibrium, the equilibrium reaches mutual **defection**. In subcase (iii)(c), we have $p_1 = q_1 = 0, p_4 = p_4 = 0$. Since the rounds alternate between CC and DD after one of these outcomes is first played, we say the equilibrium reaches **cis-alternation**.

The subcases (iii)(a) and (i)(c) are impossible by definition. In case (i)(a), we can assume that either CD or DC occurs at equilibrium. Suppose that CD does. Note that CC and DD never occur at equilibrium. If there is a deterministic transition CD→DC, then $p_2 = 0, q_3 = 1$. Since $q_2 = p_2$ and $p_3 = q_3$ by assumption, the rounds alternate between CD and DC after one of these outcomes is first played. We say the equilibrium reaches **trans-alternation**. If there is a deterministic transition CD→CD, then $p_2 = 1, q_3 = 0$. Since CD is an absorbing state played at equilibrium, we say the equilibrium reaches **CD-repetition**. (The same two possibilities arise if DC occurs at equilibrium.)

In summary, there are five possibilities for the symmetric memory-1 Nash equilibrium. For each type of equilibrium, there is a natural conventional choice of starting moves: an equilibrium of mutual cooperation starts in CC, an equilibrium of mutual defection starts in DD, an equilibrium of cis-alternation starts in CC, and equilibrium of trans-alternation starts in CD, and an equilibrium of CD-repetition starts in CD. (Some other possibilities can exist, but they are either redundant or unusual: for instance, starting in DD and immediately transitioning to mutual cooperation CC thereafter.)

Derivation of main results

By the previous subsection, there are five types of symmetric memory-1 equilibria which are deterministic along the equilibrium path. The equilibria are given by the following pairs of strategies:

1. (Cooperation) $(1; 1, p_2, p_3, p_4), (1; 1, p_2, p_3, p_4)$
2. (Defection) $(0; p_1, p_2, p_3, 0), (0; p_1, p_2, p_3, 0)$
3. (trans-Alternation) $(1; p_1, 0, 1, p_4), (0; p_1, 0, 1, p_4)$
4. (cis-Alternation) $(1; 0, p_2, p_3, 1), (1; 0, p_2, p_3, 1)$
5. (CD-Repetition) $(1; p_1, 1, 0, p_4), (0; p_1, 1, 0, p_4)$

To prove Propositions 1-2, we want to derive conditions in the variables p_i for these strategy pairs to be Nash equilibria, and we want to prove that if they are Nash equilibria, then they are also LF-stable Nash equilibria.

To investigate LF-stability, we invoke Proposition 9. We must test that neither strategy has an incentive to deviate to the memory-0 follower-type strategies ALLC, ALLD, COPY, or ACOPY.

Case 1 (Cooperation): We have the strategy pair $(\mathbf{p}, \mathbf{p})$, where $\mathbf{p} = (1; 1, p_2, p_3, p_4)$. Deviation to ALLC or to COPY by either player does not affect the actions played, so it cannot be profitable. The discounted payoff to player 1 at equilibrium is $\mathbf{w}_{\mathbf{pp}} \cdot (a, b, c, d)$, which is easily computed to be just 'a'. We want to compare this to $\mathbf{w}_{\text{ALLD}\mathbf{p}} \cdot (a, b, c, d)$ and $\mathbf{w}_{\text{ACOPY}\mathbf{p}} \cdot (a, b, c, d)$. Consider the memory-1 strategy $\mathbf{q} = (0; 0, 1, 1, 1)$. Then it is easy to check that

$$\begin{aligned} \mathbf{w}_{\mathbf{qp}} &= \alpha \mathbf{w}_{\mathbf{pp}} + (1-\alpha)\mathbf{w}_{\text{ACOPY}\mathbf{p}} \\ \alpha &= \Big(\frac{\delta((1-\delta)p_2 + \delta p_3)}{1 + \delta((1+\delta)p_3 - \delta p_2)}\Big) \in (0,1) \end{aligned} \tag{29}$$

In other words, $\mathbf{w}_{\mathbf{qp}}$ is a convex combination of $\mathbf{w}_{\mathbf{pp}}$ and $\mathbf{w}_{\text{ACOPY}\mathbf{p}}$. It follows immediately that if player 1 has a profitable deviation to ACOPY, then there is a profitable deviation to $\mathbf{q} \in \mathbf{Mem}^1$ as well. On the other hand, deviation to COPY does not affect the payoffs of either player, and ALLC and ALLD are both in $\mathbf{Mem}^1$. So if there is a profitable deviation to any follower-type strategy, then there is also a profitable deviation to a strategy in $\mathbf{Mem}^1$. This means that the conditions for Nash equilibrium and LF-stable Nash equilibrium coincide. By the discussion above, these conditions are

$$\begin{aligned} \mathbf{w}_{\mathbf{pp}} \cdot (a, b, c, d) &\geqslant \mathbf{w}_{\text{ALLD}\mathbf{p}} \cdot (a, b, c, d) \\ \mathbf{w}_{\mathbf{pp}} \cdot (a, b, c, d) &\geqslant \mathbf{w}_{\text{ACOPY}\mathbf{p}} \cdot (a, b, c, d) \end{aligned} \tag{30}$$

These inequalities are easy to express explicitly and simplify. We have given the results in Proposition 1.

Case 2 (Defection): There is no fundamental difference between the actions C and D besides a choice of label. Therefore the method in the previous case works exactly the same way for this case.

Case 3 (trans-Alternation): We have the strategy pair $(\mathbf{p}, \mathbf{q})$, where $\mathbf{p} = (1; p_1, 0, 1, p_4)$ and $\mathbf{q} = (0; p_1, 0, 1, p_4)$. Deviation to ACOPY by either player does not affect the actions played, so it cannot be profitable. It is also easy to check that

$$(\mathbf{w}_{\text{COPY}\mathbf{q}} - \mathbf{w}_{\mathbf{pq}}) = \underbrace{\Big(\frac{(1+\delta(1-p_1))p_4}{1-\delta(p_1-p_4)}\Big)}_{>0}(\mathbf{w}_{\text{ALLC}\mathbf{q}} - \mathbf{w}_{\mathbf{pq}}) + \underbrace{\Big(\frac{(1-\delta p_1)(1+\delta p_4)}{1-\delta(p_1-p_4)}\Big)}_{>0}(\mathbf{w}_{\text{ALLD}\mathbf{q}} - \mathbf{w}_{\mathbf{pq}}) \tag{31}$$

The equation above implies that any profitable deviation by player 1 from $\mathbf{p}$ to COPY also implies the existence of a profitable deviation to ALLC or ALLD, which are in $\mathbf{Mem}^1$. More generally, if there is a profitable deviation by either player to any follower-type strategy, then there is such a deviation for player 1, and then also a profitable deviation by player 1 to a strategy ALLC or ALLD in $\mathbf{Mem}^1$. In particular, the conditions for Nash equilibrium and LF-stable Nash equilibrium coincide. By the discussion above,

these conditions are

$$\begin{aligned} \mathbf{w_{pq}} \cdot (a,b,c,d) &\geqslant \mathbf{w}_{\mathrm{ALLD}\mathbf{q}} \cdot (a,b,c,d) \\ \mathbf{w_{pq}} \cdot (a,b,c,d) &\geqslant \mathbf{w}_{\mathrm{ALLC}\mathbf{q}} \cdot (a,b,c,d) \end{aligned} \tag{32}$$

These inequalities are easy to express explicitly and simplify. We have given the results in Proposition 1.

Case 4 (cis-Alternation): We have the strategy pair $(\mathbf{p}, \mathbf{p})$, where $\mathbf{p} = (1; 0, p_2, p_3, 1)$. Deviation to COPY by either player does not affect the actions played, so it cannot be profitable. It is also easy to check that

$$\begin{aligned} &(\mathbf{w}_{\mathrm{ACOPY}\mathbf{p}} - \mathbf{w_{pp}}) = \\ &\underbrace{\Big(\frac{(1-p_2)(1+\delta p_3)}{1-\delta(p_2-p_3)}\Big)}_{>0}(\mathbf{w}_{\mathrm{ALLC}\mathbf{p}} - \mathbf{w_{pp}}) + \underbrace{\Big(\frac{(1+\delta(1-p_2))(1-\delta(1-p_3))}{1-\delta(p_2-p_3)}\Big)}_{>0}(\mathbf{w}_{\mathrm{ALLD}\mathbf{p}} - \mathbf{w_{pp}}) \end{aligned} \tag{33}$$

This means, in particular, that any profitable deviation to ACOPY implies the existence of a profitable deviation to ALLC or ALLD, which are in $\mathbf{Mem}^1$. So if there is a profitable deviation to any follower-type strategy, then there is also a profitable deviation to a strategy in $\mathbf{Mem}^1$. This means the the conditions for Nash equilibrium and LF-stable Nash equilibrium coincide. By the discussion above, these conditions are

$$\begin{aligned} \mathbf{w_{pp}} \cdot (a,b,c,d) &\geqslant \mathbf{w}_{\mathrm{ALLD}\mathbf{p}} \cdot (a,b,c,d) \\ \mathbf{w_{pp}} \cdot (a,b,c,d) &\geqslant \mathbf{w}_{\mathrm{ALLC}\mathbf{p}} \cdot (a,b,c,d) \end{aligned} \tag{34}$$

These inequalities are easy to express explicitly and simplify. We have given the results in Proposition 1.

Case 5 (CD-Repetition): We have the strategy pair $(\mathbf{p}, \mathbf{q})$, where $\mathbf{p} = (1; p_1, 1, 0, p_4)$ and $\mathbf{q} = (0; p_1, 1, 0, p_4)$. Deviation to ACOPY by either player does not affect the actions played, so it cannot be profitable. The same is true of deviation to ALLC by player 1 or deviation to ALLD by player 2. The discounted payoff to player 1 at equilibrium is $\mathbf{w_{pq}} \cdot (a,b,c,d)$, which is easily computed to be just 'b'. We want to compare this to $\mathbf{w}_{\mathrm{ALLD}\mathbf{q}} \cdot (a,b,c,d)$ and $\mathbf{w}_{\mathrm{COPY}\mathbf{q}} \cdot (a,b,c,d)$. Consider the memory-1 strategy $\mathbf{r} = (0; 1, 0, 1, 1)$. Then it is easy to check that

$$\begin{aligned} \mathbf{w_{rq}} &= \beta \mathbf{w_{pq}} + (1-\beta)\mathbf{w}_{\mathrm{COPY}\mathbf{q}} \\ \beta &= \Big(\frac{\delta(1-(1-\delta)p_4 - \delta p_1))}{1-\delta((1+\delta)p_1 - (1+\delta p_4))}\Big) \in (0,1) \end{aligned} \tag{35}$$

In other words, $\mathbf{w_{rp}}$ is a convex combination of $\mathbf{w_{pq}}$ and $\mathbf{w}_{\mathrm{COPY}\mathbf{q}}$. It follows immediately that if player 1 has a profitable deviation to COPY, then there is a profitable deviation to $\mathbf{r} \in \mathbf{Mem}^1$ as well. On the other hand, deviation to ACOPY does not affect the payoffs of either player, and ALLC and ALLD are both in $\mathbf{Mem}^1$. So if there is a profitable deviation by player 1 to any follower-type strategy, then there is also a profitable deviation to a strategy in $\mathbf{Mem}^1$.

There is a similar analysis for player 2. The discounted payoff to player 2 at equilibrium is $\mathbf{w_{pq}} \cdot$

(a, c, b, d), which is easily computed to be just 'c'. We want to compare this to $\mathbf{w}_{\mathbf{p}\text{ALLC}} \cdot (a, c, b, d)$ and $\mathbf{w}_{\mathbf{p}\text{COPY}} \cdot (a, c, b, d)$. Consider the memory-1 strategy $\mathbf{s} = (1; 0, 0, 1, 0)$. Then it is easy to check that

$$\begin{aligned} \mathbf{w}_{\mathbf{ps}} &= \gamma \mathbf{w}_{\mathbf{pq}} + (1-\gamma)\mathbf{w}_{\mathbf{p}\text{COPY}} \\ \gamma &= \Big(\frac{\delta((1-\delta)p_1 + \delta p_4)}{1 + \delta((1+\delta)p_4 - \delta p_1)}\Big) \in (0,1) \end{aligned} \tag{36}$$

In other words, $\mathbf{w}_{\mathbf{ps}}$ is a convex combination of $\mathbf{w}_{\mathbf{pq}}$ and $\mathbf{w}_{\mathbf{p}\text{COPY}}$. It follows immediately that if player 2 has a profitable deviation to COPY, then there is a profitable deviation to $\mathbf{s} \in \mathbf{Mem}^1$ as well. On the other hand, deviation to ACOPY does not affect the payoffs of either player, and ALLC and ALLD are both in $\mathbf{Mem}^1$. So if there is a profitable deviation by player 2 to any follower-type strategy, then there is also a profitable deviation to a strategy in $\mathbf{Mem}^1$.

The discussion above means that the conditions for Nash equilibrium and LF-stable Nash equilibrium coincide. We have shown these conditions are

$$\begin{aligned} \mathbf{w}_{\mathbf{pq}} \cdot (a, b, c, d) &\geqslant \mathbf{w}_{\text{ALLD}\mathbf{q}} \cdot (a, b, c, d) \\ \mathbf{w}_{\mathbf{pq}} \cdot (a, b, c, d) &\geqslant \mathbf{w}_{\text{COPY}\mathbf{q}} \cdot (a, b, c, d) \\ \mathbf{w}_{\mathbf{pq}} \cdot (a, c, b, d) &\geqslant \mathbf{w}_{\mathbf{p}\text{ALLC}} \cdot (a, c, b, d) \\ \mathbf{w}_{\mathbf{pq}} \cdot (a, c, b, d) &\geqslant \mathbf{w}_{\mathbf{p}\text{COPY}} \cdot (a, c, b, d) \end{aligned} \tag{37}$$

These inequalities are easy to express explicitly and simplify. We have given the results in Proposition 1.

In summary, we have given proofs of Propositions 1-2 here. Note that Propositions 3 and 4 are simple corollaries which follow from elementary algebra.

Equalizers

For an arbitrary strategy $\mathbf{p} = (p_0; p_1, p_2, p_3, p_4)$ we calculate the following:

$$\mathbf{w}_{\text{ALLC}\mathbf{p}} = \Big(\Big(\frac{(1-\delta)p_0 + \delta p_3}{1 - \delta(p_1 - p_3)}\Big), \Big(\frac{1 - (1-\delta)p_0 - \delta p_1}{1 - \delta(p_1 - p_3)}\Big), 0, 0\Big) \tag{38}$$

$$\mathbf{w}_{\text{ALLD}\mathbf{p}} = \Big(0, 0, \Big(\frac{(1-\delta)p_0 + \delta p_4}{1 - \delta(p_2 - p_4)}\Big), \Big(\frac{1 - (1-\delta)p_0 - \delta p_2}{1 - \delta(p_2 - p_4)}\Big)\Big) \tag{39}$$

$$\mathbf{w}_{\text{COPY}\mathbf{p}} = \Big(\Big(\frac{(1-\delta)p_0 + \delta p_4}{1 - \delta(p_1 - p_4)}\Big), 0, 0, \Big(\frac{1 - (1-\delta)p_0 - \delta p_1}{1 - \delta(p_1 - p_4)}\Big)\Big) \tag{40}$$

$$\mathbf{w}_{\text{ACOPY}\mathbf{p}} = \Big(0, \Big(\frac{1 - (1-\delta)p_0 - \delta p_2}{1 - \delta(p_2 - p_3)}\Big), \Big(\frac{(1-\delta)p_0 + \delta p_3}{1 - \delta(p_2 - p_3)}\Big), 0\Big) \tag{41}$$

For any 2×2 payoff matrix, a best reply to $\mathbf{p}$ can be found among the set $\{\text{ALLC}, \text{ALLD}, \text{COPY}, \text{ACOPY}\}$. This means that if the payoffs for all four strategies are equal, then every follower-type strategy earns an identical payoff against $\mathbf{p}$. Such a strategy would be a **leader-follower equalizer**, which is a generalization of an ordinary equalizer (for which every ordinary strategy earns an identical payoff against $\mathbf{p}$).

The defining equations for an LF-equalizer are then

$$\mathbf{w}_{\text{ALLC}\mathbf{p}} \cdot (a,b,c,d) = \mathbf{w}_{\text{ALLD}\mathbf{p}} \cdot (a,b,c,d) = \mathbf{w}_{\text{COPY}\mathbf{p}} \cdot (a,b,c,d) = \mathbf{w}_{\text{ACOPY}\mathbf{p}} \cdot (a,b,c,d) \tag{42}$$

However, it is conventionally insisted (5) that the equalizing property should also be independent of p_0. We insist the same for an LF-equalizer. Therefore, we take derivatives of the equalities (42) with respect to p_0 and simplify. The result is

$$\frac{c-d}{1-\delta(p_2-p_4)} = \frac{a-b}{1-\delta(p_1-p_3)} = \frac{a-d}{1-\delta(p_1-p_4)} = \frac{c-b}{1-\delta(p_2-p_3)} \tag{43}$$

Conversely, it is not hard to check that (43) also implies (42) for all p_0. So (43) are the defining equations for LF-equalizers. The system contains some redundancy. Specifically, the last equality is redundant. If $a-d=0$, then the first two equalities imply the last immediately since all numerators must be 0. On the other hand, if $a-d \neq 0$, then the first two equalities can be solved for p_2, p_3 in terms of p_1, p_4 and plugged into the last expression to prove the final equality. Indeed, solving for p_2, p_3 in this way recovers exactly the expressions for p_2 and p_3 for an ordinary equalizer strategy (5). This means that ordinary equalizers are also LF-equalizers: the two concepts coincide. An interesting consequence is that an equalizer strategy can always announce its next move publicly and still successfully equalize every opponent.

Appendix: Mathematical tools

Markov chains

Some textbook references for basic Markov chains are (6; 1). For our purposes, "Markov chain" means "finite Markov chain" unless otherwise specified.

A Markov chain is a random process described by a set of states $S = \{s_1, \ldots, s_n\}$, together with a transition probability $p(i, j)$. The set S is called the state space. At each time $t = 0, 1, 2, \ldots$ the chain is in some state $s_i \in S$. The next state—that is, the state at time $t + 1$—will be s_j with probability $p(i, j)$.

A probability distribution on the set of states is a vector $\mathbf{v} = (v_1, \ldots, v_n)$, with $v_i \in [0, 1]$ and $\sum_i v_i = 1$. For example, suppose $\mathbf{v}_t = (v_1, \ldots, v_n)$ is a probability distribution describing the state of the chain at time t. This means that v_i is the probability of being in state s_i at time t. Then the state of the chain at time $t + 1$ is described by the vector $\mathbf{v}_{t+1} = \mathbf{v}_t\mathbf{M}$, where $\mathbf{M}$ is an $n \times n$ matrix called the transition matrix of the chain. The (i, j)th entry of $\mathbf{M}$ is just $p(i, j)$.

An initial distribution $\mathbf{v}_0$ is a probability distribution describing the state at time $t = 0$. The distribution $\mathbf{v}_t = \mathbf{v}_0\mathbf{M}^t$ describes the probabilities of being in each state at time t.

Definition 1. *A subset of states $S' \subseteq S$ is called **closed** if for every $s_i \in S'$ and $s_j \notin S'$, we have $p(i, j) = 0$. A state $s \in S$ is called an **absorbing state** if $\{s\}$ is closed.*

Markov decision processes

A Markov decision process (MDP) generalizes the notion of a Markov chain by adding a decision maker. Formally, a finite MDP consists of a finite set $S = \{s_1, \ldots, s_n\}$ of states, a finite set A of actions, a transition probability $p_a(i, j)$, and a reward function $r_a(i)$. At each time $t = 0, 1, 2, \ldots$ the chain is in some state $s_i \in S$. The decision maker chooses some action a and collects an immediate reward $r_a(i)$. The next state will be s_j with probability $p_a(i, j)$.

A strategy σ for the MDP describes which action to take based on the history of the process. Formally, let $\mathbf{h}$ be a history, that is, an ordered list of states visited and actions taken up to the present time t. Then $\sigma(\mathbf{h}, a)$ represents the probability to take action a in the current round. If $\sigma(\mathbf{h}, a) \in \{0, 1\}$ for all histories $\mathbf{h}$ and actions a, then the strategy is deterministic.

A stationary strategy π is a strategy which chooses an action only based on the current state. So we write $\pi(i, a)$ for the probability to take action a if the current state is s_i.

The following is a useful and well-known fact (7; 8):

Proposition 10. *An MDP has a deterministic optimal stationary strategy. That is, there is a deterministic stationary strategy π whose discounted reward is at least as much as the discounted reward achieved by any other strategy σ, regardless of the starting state.*

The discounted reward for an MDP is calculated exactly the same way as the discounted payoff in a

repeated game: the reward in round i is weighted by a factor of δ^{i-1}, and the sum of discounted rewards is multiplied by a factor of $(1-\delta)$. The expected value of this quantity is the discounted reward.

References

[1] Rick Durrett. *Probability: Theory and Examples*. Cambridge University Press, Cambridge, UK, 5th edition, 2019.

[2] René Levínskỳ, Abraham Neyman, and Miroslav Zelenỳ. Should i remember more than you? best responses to factored strategies. *International Journal of Game Theory*, 49(4):1105–1124, 2020.

[3] Pedro Dal Bó and Guillaume R Fréchette. The evolution of cooperation in infinitely repeated games: Experimental evidence. *American Economic Review*, 101(1):411–429, 2011.

[4] Maarten C Boerlijst, Martin A Nowak, and Karl Sigmund. Equal pay for all prisoners. *The American mathematical monthly*, 104(4):303–305, 1997.

[5] Genki Ichinose and Naoki Masuda. Zero-determinant strategies in finitely repeated games. *Journal of theoretical biology*, 438:61–77, 2018.

[6] Sheldon M Ross. *Introduction to Probability Models*. Academic Press, San Diego, CA, 10th edition, 2010.

[7] David Blackwell. Discrete dynamic programming. *The Annals of Mathematical Statistics*, pages 719–726, 1962.

[8] Lodewijk Kallenberg. Markov decision processes. *Lecture Notes. University of Leiden*, 428, 2011.
